# Alternative splicing of the SUMO1/2/3 transcripts affects cellular SUMOylation and produces functionally distinct SUMO protein isoforms

**DOI:** 10.1038/s41598-023-29357-7

**Published:** 2023-02-09

**Authors:** Myriah L. Acuña, Andrea García-Morin, Rebeca Orozco-Sepúlveda, Carlos Ontiveros, Alejandra Flores, Arely V. Diaz, Isabel Gutiérrez-Zubiate, Abhijeet R. Patil, Luis A. Alvarado, Sourav Roy, William K. Russell, Germán Rosas-Acosta

**Affiliations:** 1 Department of Biological Sciences, The University of Texas at El Paso, El Paso, TX 79968 USA; 2 Department of Genetics, University of Pennsylvania Perelman School of Medicine, Philadelphia, PA 19104 USA; 3 Biostatistics and Epidemiology Consulting Lab, Texas Tech University Health Sciences Center, El Paso, TX 79905 USA; 4 Border Biomedical Research Center, University of Texas at El Paso, El Paso, TX 79968 USA; 5 Department of Biochemistry and Molecular Biology, The University of Texas Medical Branch, Galveston, TX 77555 USA; 6 Present Address: Graduate School of Biomedical Sciences, University of Texas Health, San Antonio, TX 78229 USA; 7 Present Address: Department of Pathology, Microbiology, and Immunology, Vanderbilt University Medical Center, Nashville, TN 37232 USA; 8 Present Address: Department of Medicine, University of Colorado Anschutz Medical Campus, Aurora, CO 80045 USA

**Keywords:** Sumoylated proteins, RNA splicing, Post-translational modifications, Sumoylation, RNA transport

## Abstract

Substantial increases in the conjugation of the main human SUMO paralogs, SUMO1, SUMO2, and SUMO3, are observed upon exposure to different cellular stressors, and such increases are considered important to facilitate cell survival to stress. Despite their critical cellular role, little is known about how the levels of the SUMO modifiers are regulated in the cell, particularly as it relates to the changes observed upon stress. Here we characterize the contribution of alternative splicing towards regulating the expression of the main human SUMO paralogs under normalcy and three different stress conditions, heat-shock, cold-shock, and Influenza A Virus infection. Our data reveal that the normally spliced transcript variants are the predominant mature mRNAs produced from the SUMO genes and that the transcript coding for SUMO2 is by far the most abundant of all. We also provide evidence that alternatively spliced transcripts coding for protein isoforms of the prototypical SUMO proteins, which we refer to as the SUMO alphas, are also produced, and that their abundance and nuclear export are affected by stress in a stress- and cell-specific manner. Additionally, we provide evidence that the SUMO alphas are actively synthesized in the cell as their coding mRNAs are found associated with translating ribosomes. Finally, we provide evidence that the SUMO alphas are functionally different from their prototypical counterparts, with SUMO1α and SUMO2α being non-conjugatable to protein targets, SUMO3α being conjugatable but targeting a seemingly different subset of protein from those targeted by SUMO3, and all three SUMO alphas displaying different cellular distributions from those of the prototypical SUMOs. Thus, alternative splicing appears to be an important contributor to the regulation of the expression of the SUMO proteins and the cellular functions of the SUMOylation system.

## Introduction

SUMOylation, the covalent attachment of a Small Ubiquitin-like MOdifier (SUMO) to a protein target, involves four different enzymatic steps. First, the SUMO molecule must be proteolytically processed by SUMO peptidases/isopeptidases to cleave-off a short C-terminal sequence, thus exposing an internal di-Gly sequence that becomes the carboxyl end of the mature SUMO protein (i.e., the proteolytically processed form). Second, SUMO is activated in an ATP-dependent manner by SAE2/SAE1, the SUMO Activating Enzyme heterodimer. Activation results in SUMO forming sequential thioester bonds through its carboxyl di-Gly sequence, first with SAE2/SAE1 and subsequently with the SUMO conjugating enzyme, Ubc9. Third, SUMO is target-conjugated via the formation of an isopeptide bond with the ε-amino group of a Lys residue in the target protein, a process catalyzed by Ubc9. This step is frequently enhanced by the action of a SUMO ligase, which constitutes the fourth enzymatic activity involved in the pathway. SUMO ligases facilitate the formation of the isopeptide bond and provide some specificity to the process, as SUMO ligases are active over a relatively narrow range of protein targets. SUMOylated targets can subsequently become de-SUMOylated through the isopeptidase activity of de-SUMOylating enzymes. While there are only single SUMO activating and conjugating enzymes, there are numerous SUMO ligases and peptidases/isopeptidases. As for the actual SUMO modifier, there are five SUMO modifiers in humans, namely SUMO1, SUMO2, SUMO3, SUMO4, and SUMO5, each encoded by a separate gene (reviewed in^1–6^).

The *SUMO* genes likely arose via successive gene duplication events, as deduced from their phylogenetic analysis and exon/intron structure^7,8^. The first duplication produced the primordial *SUMO*1/5 and *SUMO*2/3/4 genes. The primordial *SUMO*2/3/4 gene underwent one gene duplication that generated the precursor for *SUMO4* and the primordial *SUMO*2/3 gene, and the primordial *SUMO*2/3 gene duplicated again to generate the precursors for the current *SUMO2* and *SUMO3* genes. Similarly, the primordial *SUMO*1/5 gene underwent one additional gene duplication that over time generated the current *SUMO1* and *SUMO5* genes. Each gene duplication provided some freedom from the selective constraints related to the function of the primordial copy, thus allowing the functional differentiation and divergence that resulted in the five *SUMO* genes presently found in the human genome. The proteins encoded by these genes exhibit very similar overall shapes, variable levels of amino acid identity, and clear functional differentiation, as recently demonstrated^9^. As such, the *SUMO* genes and their protein products can be considered to be paralogs, as per current definition of the term^10,11^. In their mature proteolytically-processed form, out of the five SUMO paralogs present in humans, SUMO2 and SUMO3 exhibit the closest sequence identity, differing from each other only by three amino acid residues. SUMO1 exhibits only 49% identity with SUMO2. SUMO4 is more closely related to SUMO2/3 than to SUMO1, exhibiting 85% identity to SUMO2. Finally, SUMO5 is more closely related to SUMO1 than to SUMO2/3, displaying 88% identity with SUMO1. Importantly, SUMO1, 2, and 3 are widely expressed throughout the body, with their transcripts being easily detected in most organs and tissues^9^. In contrast, SUMO4 expression is limited to kidney, immune cells, pancreas, and placenta^12,13^, and SUMO5 is limited to blood cells and testis^9,14^.

SUMOylation regulates every major event taking place in mammalian cells, including DNA repair^15,16^, transcription^17,18^, splicing^19^, ribosomal assembly^20^, progression through the cell cycle^21^, mitosis^22^, meiosis^23^, nucleocytoplasmic traffic^24^, signal transduction^25^, cytoskeletal and mitochondrial dynamics^26,27^, apoptosis and autophagy^28–31^, the activation of ion channels^32^, glycolysis^33,34^, and every metabolic pathway^35^. In addition to its critical role as a regulator of normal cellular functions, SUMOylation also coordinates the adaptive responses required to survive most cellular stressors, including genotoxic attack^36,37^, heat-shock^38^, cold-shock^39^, oxygen and glucose deprivation^40–42^, and viral infection^43,44^. Importantly, all the stresses enumerated above result in substantial increases in the overall profile of SUMO conjugation in the cell, a phenomenon best observed by immunoblot analysis.

The mechanisms responsible for the global increases in cellular SUMOylation triggered by stress remain to be fully characterized. The initial reports related to an increase in cellular SUMOylation during stress indicated that only SUMO2 and SUMO3 SUMOylation were increased. However, subsequent reports by us and others indicated that, for some types of stress, the increase in cellular SUMOylation also involved SUMO1^40,45,46^. As for how the increase in SUMOylation is achieved, some authors have indicated, based primarily on assessments performed using mass spectrometry data, that the increases are the result of a redistribution of SUMO from one pool of targets, including free unconjugated SUMO, to another^38,47^. Such redistribution could be mediated by the activation and/or inactivation of specific sets of SUMO deconjugating enzymes and SUMO ligases. This redistribution model precludes the need for a net increase in the expression of any given SUMO paralog.

While the redistribution of SUMO from one pool of targets to another is unquestionably involved in the SUMO-mediated responses to stress, findings by us and other groups support the need for additional SUMO synthesis as a likely part of the process. First, using a serial dilution approach in conjunction with immunoblot detection, we estimated the increase in global cellular SUMOylation triggered by Influenza A Virus (IAV) infection to be about twofold (i.e., 100%)^46^. This increase is unlikely to result from a simple redistribution of SUMO, as it involved SUMO1, a paralog that is found mostly in the conjugated form, with a very limited pool of free SUMO and a substantial fraction conjugated to RanGAP and therefore protected from isopeptidases^48^. Second, an unbiased proteomic analysis of endogenous SUMOylation upon heat-shock in HEK293 cells found that the stress-induced increase in SUMO2/3-SUMOylation likely required ongoing SUMO2/3 synthesis, as the pool of free SUMO2/3 was only ~ 6%^49^. Third, a study performed using U2OS and HEK293T cells found that treatment with either of two translation inhibitors, cycloheximide and puromycin, prevented the heat-shock triggered increase in SUMO2/3 SUMOylation^50^. Finally, quantitative assessments of SUMO1 before and after exposure to hypoxia in mice showed clear net increases in SUMO1 protein and SUMO1 transcripts in the brain and heart of mice upon exposure to hypoxia^51^. Therefore, it is likely that, at least for some types of stress, and for some cells and tissues, net increases in overall cellular SUMO levels may be required for the global increases in SUMOylation observed upon stress. Such increases could be mediated by the additive effects of transcriptional, post-transcriptional, translational, and post-translational regulatory mechanisms.

Given the critical role that the global increase in cellular SUMOylation plays in conferring resistance to IAV infection (manuscript in preparation), we aimed to better characterize the post-transcriptional mechanisms involved in SUMO regulation. To this end, we first focused on alternative splicing, as there were no reports addressing this process for the SUMO genes. Alternative splicing largely increases the coding potential of the genome and correlates well with biological complexity^52^. Humans exhibit the largest prevalence of alternative splicing, with 95% of all human genes undergoing alternatively splicing^53^. Importantly, alternative splicing has been widely recognized to constitute a critical response mechanism to stress in plants^54^, and recent reports indicate that it may also play a similar role in animals, including mammals^55–57^. SUMOylation has been known to affect splicing by directly modifying numerous spliceosomal components and modulating the assembly of the spliceosome on a pre-mRNA substrate^19,58^. However, whether alternative splicing affects the cellular SUMOylation system or contributes to its overall regulation remains unknown.

Here we characterize the contribution of alternative splicing toward regulating the cellular levels of the main human SUMO paralogs, SUMO1, SUMO2, and SUMO3, under normalcy, heat-shock, cold-shock, and IAV infection. Our data indicate that SUMO2 is the predominant SUMO paralog present in the cells studied and that the normally spliced transcripts derived from the three SUMO paralogs studied constitute the predominant SUMO transcripts present in the cell. Furthermore, the cellular stressors studied trigger stress- and cell-specific changes in the profiles of alternative splicing and nuclear export of the transcripts. Importantly, our studies support the existence of a set of SUMO isoforms in the cell, which we refer to as the SUMO alpha proteins, encoded by alternatively spliced mRNA variants. Out of the SUMO alphas, SUMO1α and SUMO2α appear non-conjugatable, SUMO3α is conjugatable, and all of them appear functionally distinct from their prototypical counterpart and capable of exhibiting regulatory functions for the SUMOylation system. Future studies aimed at better understanding the roles played by the SUMO alphas are likely to provide critical information toward achieving the full therapeutical potential of SUMO-targeted clinical interventions.

## Results

### The three main SUMO paralogs, SUMO1, SUMO2, and SUMO3, are alternatively spliced producing variant transcripts coding for one additional protein isoform for every paralog

While substantial progress has been achieved in characterizing the functions and effects associated with SUMOylation, our knowledge of the mechanisms regulating the activity of the SUMOylation system remains limited. One particular area that remains unexplored is the potential contribution that post-transcriptional processing may play in regulating cellular SUMOylation. To address this knowledge gap, we explored the NCBI database in search of previously identified alternatively spliced transcripts for the three main SUMO paralogs expressed in humans, namely SUMO1, SUMO2, and SUMO3. SUMO4 and SUMO5 were not considered given their restricted expression and poorly characterized function.

**Figure 1:**
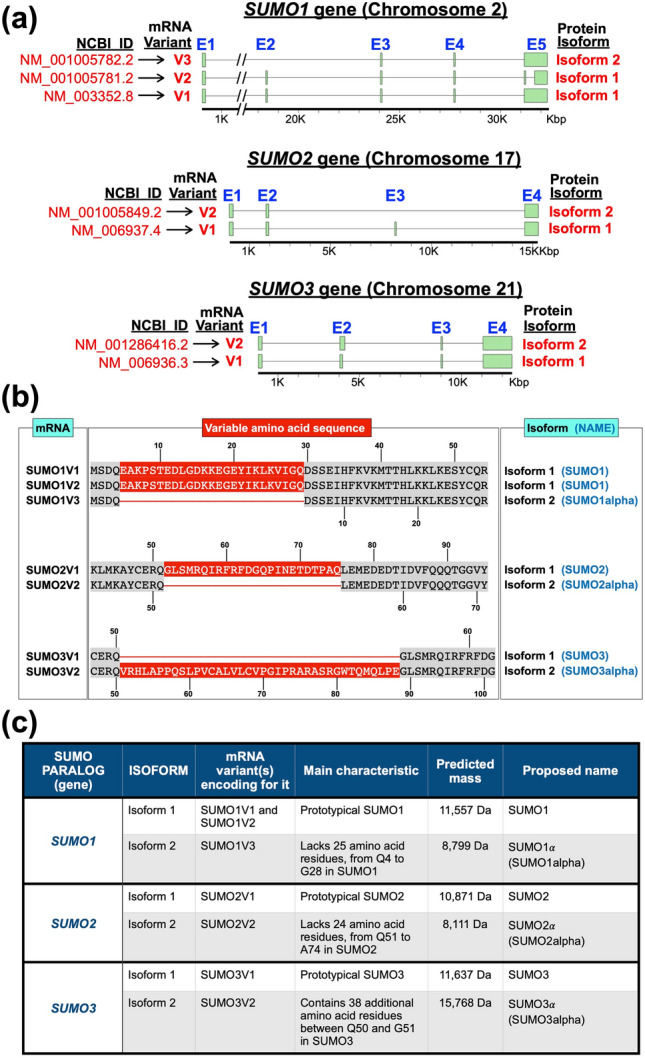
Predominant variant forms of the mature mRNAs coding for the three main SUMO paralogs in humans, according to current NCBI RNA-sequencing data. (**a**) Schematic of the SUMO1, SUMO2, and SUMO3 genes, showing the distribution of exons (E) (green rectangles) and introns (connecting lines) along the genes and the different transcripts produced by normal and alternative splicing. The bars at the bottom represent the gene, with residues from the transcriptional start site indicated in thousands of base pairs (Kbp). The NCBI identifier and name given to each variant are indicated to the left (in red) and the protein isoform encoded by each variant is indicated to the right. In every case, isoform 1 is the prototypical SUMO protein. (**b**) Schematic emphasizing the specific amino acid differences (shown in red) between the proteins encoded by the normally spliced mRNA variants and the alternatively spliced ones. The numbers on top indicate the amino acid residue in the prototypical SUMO isoform (isoform 1), whereas the numbers at the bottom indicate the amino acid residues in the alternative protein isoforms, which are thereafter referred to as the SUMO alpha isoforms. (**c**) Summary of the properties of the different protein isoforms encoded by each SUMO paralog, including their proposed names, the specific transcript variant(s) coding for them, and their main features.

The RNA-seq data deposited in the NCBI database provided evidence of the existence of three main mature transcripts for SUMO1, two for SUMO2, and two for SUMO3 (Fig. 1a). The mature transcripts identified are hereafter referred to as variants (abbreviated as V). Variant 1 (V1) corresponds to the normally spliced transcript, whereas the other variants correspond to alternatively spliced products. For every SUMO gene, one of the reported variants was predicted to code for a protein isoform whose primary structure differed from that of the prototypical SUMO protein. For simplicity, the predicted protein isoforms, which have not been previously reported, will be referred to as the SUMO alpha isoforms. SUMO1α and SUMO2α are encoded by mRNA variants lacking specific exons, exon 2 for SUMO1α and exon 3 for SUMO2α. Therefore, compared to their prototypical SUMO counterpart, SUMO1α and SUMO2α exhibit amino acid deletions within their primary sequence (Fig. 1a,c). In contrast, SUMO3α is encoded by an mRNA variant resulting from a splicing event that bypasses the splicing donor sequence located at the 3’ end of Exon 2. Therefore, SUMO3α contains an intronic extension to Exon 2 that adds 38 extra amino acids to its sequence, as compared with the SUMO3 (Fig. 1a,b). Notice that the splicing event that produces SUMO1V2 occurs after the stop codon located in Exon 5 and therefore does not alter the protein-coding sequence. Thus, both SUMO1V1 and SUMO1V2 code for the prototypical SUMO1 protein.

A summary of the proteins encoded by the SUMO variants characterized in this report, together with their main characteristics, is provided in Fig. 1c.

### For all SUMO paralogs analyzed, the normally spliced transcript coding for the prototypical SUMO isoform constitutes the most abundant transcript

**Figure 2:**
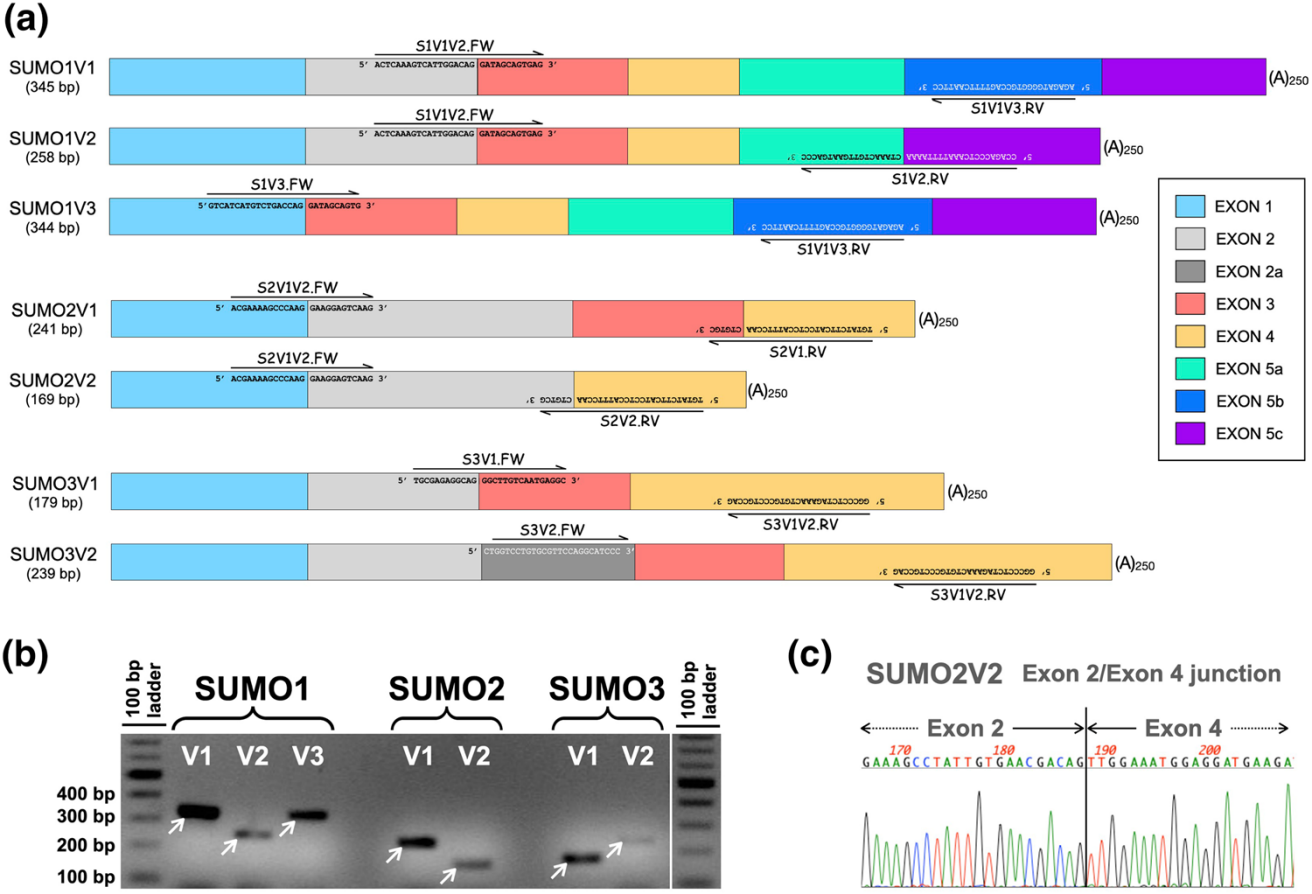
Differential detection of the predominant mRNA variant forms for SUMO1, SUMO2, and SUMO3 by RT-qPCR analyses. (**a**) Schematic of the primer pairs used to specifically amplify each mRNA variant for SUMO1, SUMO2, and SUMO3. Most of the primers correspond to exon-exon junctions, thus providing selectivity for specific splicing events; the precise location of the exon junctions in the primers, their sequence, and orientation are indicated. Reverse primers are written upside down. The length of the PCR product amplified by every primer pair is shown under the name of the variant (left, in base pairs [bp]). (A)_250_: Polyadenyl tail. (**b**) RT-qPCR products obtained for each mRNA variant form using the primer pairs represented in A. All products obtained were sequenced to confirm their identity and the specificity of the reaction. (**c**) Sequence analysis of the RT-qPCR product for SUMO2V2 showing the alternative splicing event that results in the juxtaposition of Exon 2 and Exon 4, as expected for SUMO2V2. Similar analyses confirming all predicted Exon-Exon junctions were performed for all RT-qPCR products.

To assess the contribution of each variant to the total pool of transcripts derived from each SUMO gene, we used an RT-qPCR approach. To this end, we designed primer pairs for the specific amplification of each variant. While most of the primers chosen targeted exon-exon junctions, two of the primers targeted regions fully contained within single exons (Fig. 2a). The predicted RT-qPCR products ranged in size from 169 bp for the smallest (for SUMO2V2) up to 345 bp for the largest (for SUMO1V1). RT-qPCR reactions using total RNA isolated from HEK293A cells were used to validate the primers selected. The size of the PCR products obtained, as determined by agarose gel electrophoresis, and their DNA sequence confirmed the specificity of the primer pairs chosen for every variant (Fig. 2b,c).

**Figure 3:**
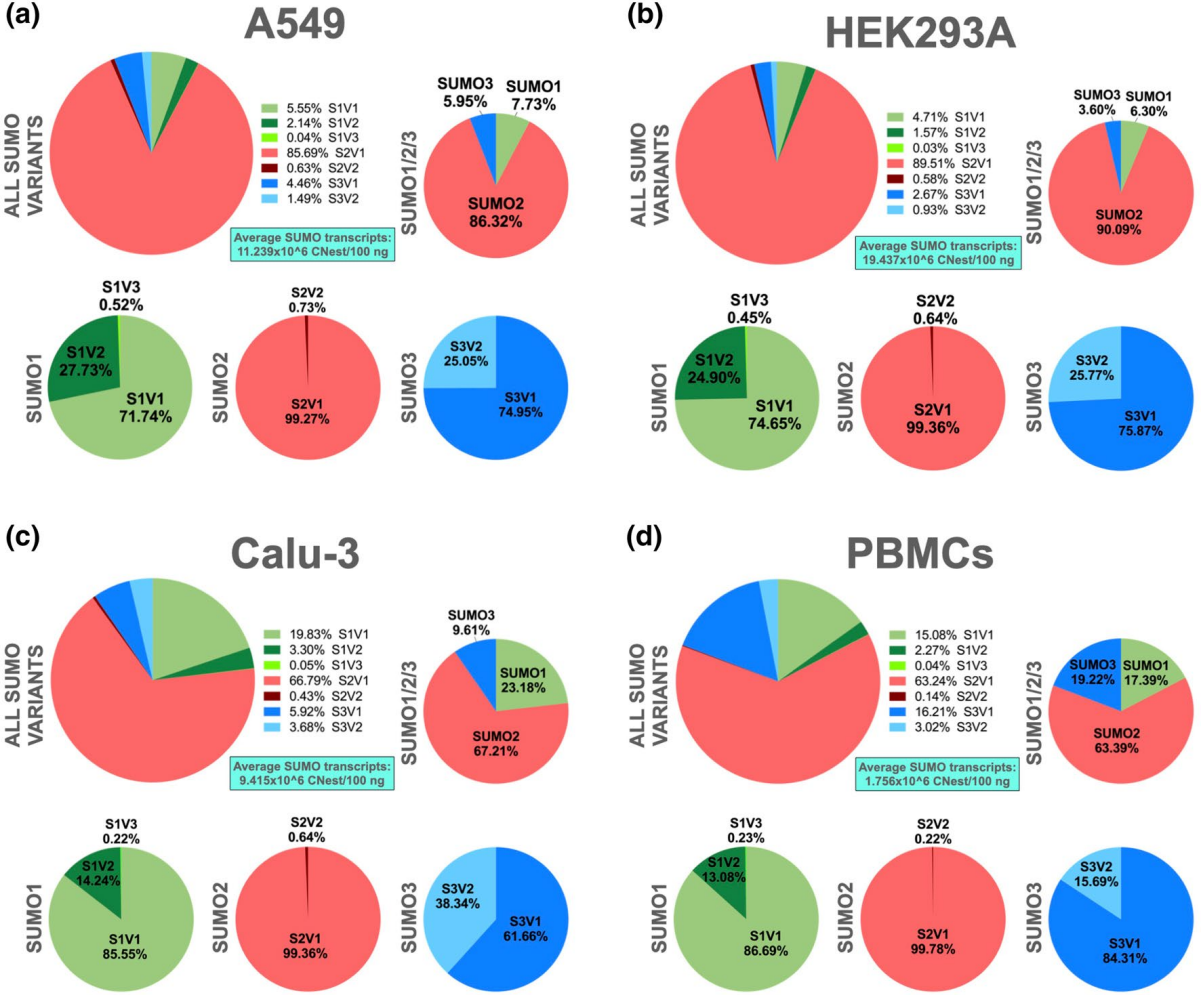
In human PBMCs and in three different cell lines of human origin, the normally spliced transcripts represent the most abundant SUMO transcripts, with SUMO2V1 constituting the most abundant of all. RT-qPCR analyses were performed as described in Materials and Methods to assess the Copy Number estimates (CNest) for each SUMO variant, using the previously validated primers described in **Fig. ****2**. The resulting average values for each transcript were added together to calculate the total CNest for all SUMO transcripts, as well as for each SUMO paralog. Using these numbers, the respective percentages corresponding to each transcript in relation to either, all SUMO transcripts (ALL SUMO VARIANTS) or to the total transcripts for its specific SUMO paralog (SUMO1, SUMO2, and SUMO3), were subsequently estimated. The calculated average CNest values for all SUMO transcripts per 100 ng of total RNA (Average SUMO transcripts) are shown for each cell type. (**a**) A549 cells. (**b**) HEK293A cells. (**c**) Calu-3 cells. (**d**) Peripheral blood mononuclear cells (PBMCs) derived from normal donors. Samples from three different donors were used in these analyses. All data represents the average values obtained from triplicate measurements in three independent experiments. For the corresponding data represented as bar graphs with error lines, see Supplementary Fig. S1. Notice that in this figure and in all subsequent figures, the labeling used to indicate the various SUMO variants has been simplified, so instead of spelling out the SUMO paralog, it is simply indicated as S, so SUMO1V1 is shown as S1V1, SUMO1V2 is shown as S1V2, and so forth.

Having validated each primer pair, we performed calibration curves using serial tenfold dilutions of in vitro transcribed RNA templates corresponding to the variant specific for each primer pair. The calibration curves obtained were subsequently used to calculate the copy number estimate (CNest) for every variant per 100 ng of total RNA. Using this approach, we estimated the average CNest for every variant in three different cell lines, namely A549 cells, HEK293A cells, and Calu-3 cells, as well as in peripheral blood mononuclear cells (PBMCs) derived from de-identified normal human donors (Fig. 3).

In all cell types assessed, the predominant SUMO transcript was SUMO2V1, ranging in abundance from a low of ~ 63% in PBMCs up to a high of ~ 90% in HEK293A cells. The second most abundant SUMO transcript was SUMO1V1, ranging from a low of ~ 5% in HEK293A cells up to a high of ~ 20% in Calu-3 cells. The only cell type displaying a different second most abundant SUMO transcript was PBMCs, in which SUMO3V1 constituted ~ 16% of transcripts, whereas SUMO1V1 represented ~ 15%. The third most abundant SUMO transcript was SUMO3V1, ranging from a low of ~ 3% in HEK293A cells up to a high of ~ 16% in PBMCs. Thus, the variants coding for the prototypical SUMO isoforms constitute the most abundant SUMO transcripts in the cells analyzed. In contrast, the least represented transcripts in all cell types were those coding for the SUMO alpha isoforms. Out of those transcripts, the one coding for SUMO3α (SUMO3V2) was the best represented, ranging from a low of ~ 1% in HEK293A cells up to a high of ~ 4% in Calu-3 cells. SUMO1V3, coding for SUMO1α, was the least abundant of all SUMO transcripts in all the cell types tested, not representing more than about 0.05% of all transcripts in any cell type (Fig. 3).

### The abundance of the different SUMO variants is affected by stress conditions in a stress-type and cell-type specific manner.

Different types of stress result in substantial increases in global cellular SUMOylation. To determine whether such increases are associated with altered splicing of the SUMO transcripts, we exposed A549 cells and HEK293A cells to different stress conditions known to trigger global increases in cellular SUMOylation and determined the CNest for each SUMO variant upon stress. Specifically, we used three different stress conditions: heat-shock (43 °C for 1 h), cold-shock (27 °C for 24 h), and influenza A virus (IAV) infection (using the A/PR/8/34 H1N1 strain at a multiplicity of infection [MOI] of 10 and collecting the cells at 12 h post-infection). To obtain reliable assessments of the changes in transcript abundance triggered by each stress condition, for every treatment performed we also measured the CNest of each SUMO variant in control cells plated at the same cell densities and maintained for the same amount of time under the absence of stress (no viral infection and normal growth temperature, i.e., 37 °C). Additionally, to ensure that the stress treatments triggered the expected cellular responses, for each stress condition we included RT-qPCR analyses performed using previously validated primer sets targeting transcripts known to be increased by that specific stress treatment (Supplementary Fig. S2). Finally, to assess the overall changes in global cellular SUMOylation, cells exposed to identical stress conditions were collected and processed for immunoblot analyses using antibodies against SUMO1 and SUMO2/3.

**Figure 4:**
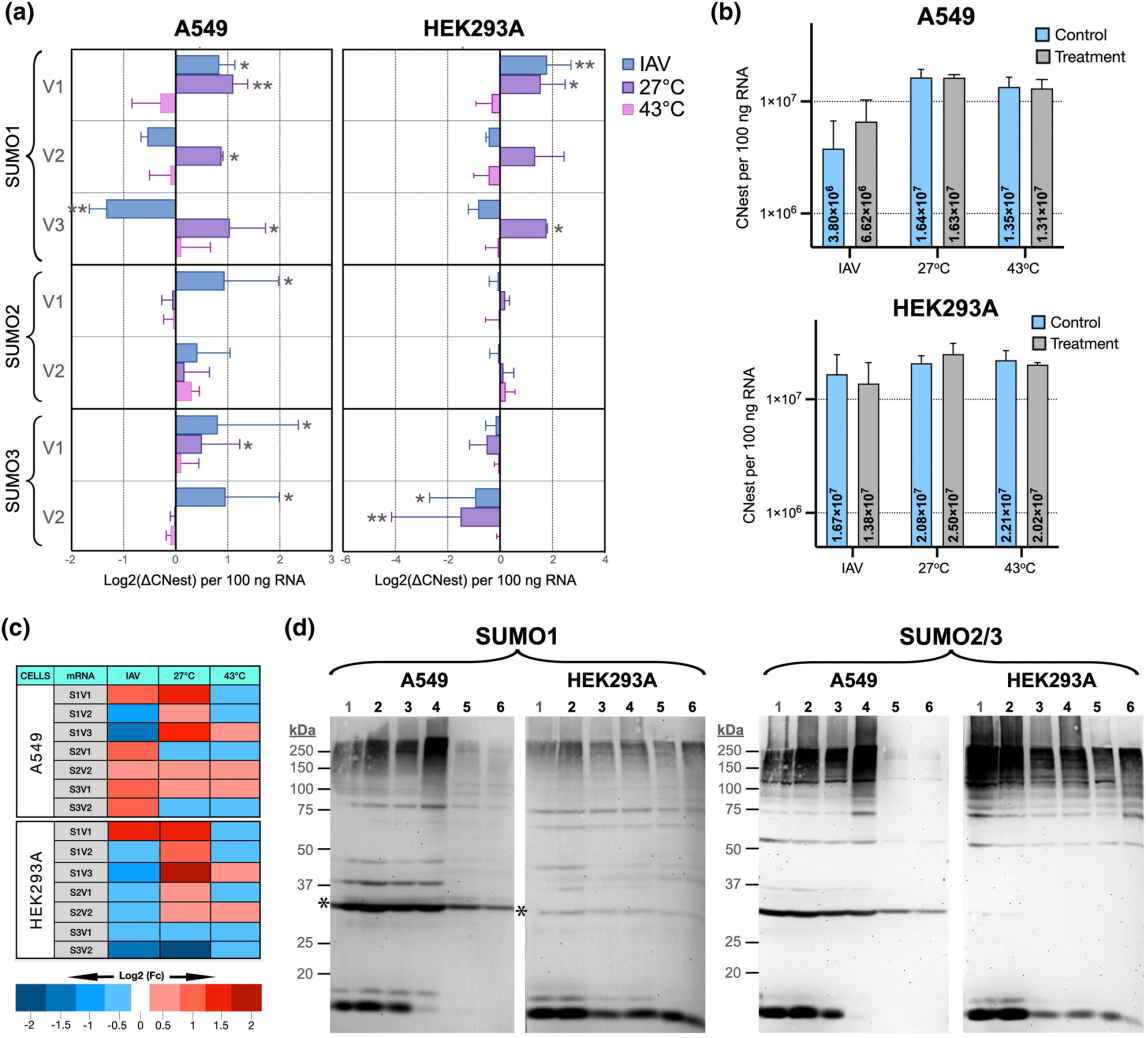
Changes in the relative abundance of the SUMO variants triggered by three different types of stress in A549 and HEK293A cells. A549 cells and HEK293A cells were exposed to three different types of stress, namely IAV (Influenza A virus infection using viral strain A/PR/8/34 H1N1 at MOI = 10 for 12 h), cold-shock (27 °C for 24 h), and heat-shock (43 °C for 1 h), as described under Materials and Methods. Untreated cell cultures plated at equal cell densities and maintained for equal time frames were used as controls. After treatment, total RNA was purified and CNest for each SUMO variant was determined. Alternatively, cells were lysed in 4 × Sample Buffer and processed for SDS-PAGE and immunoblotting. (**a**) Changes in the relative abundance of each SUMO variant, calculated by dividing the CNest value obtained upon stress by the CNest in the control (non-stressed sample). All data are shown in Log2 scale and represent the average values obtained from triplicate measurements in three independent experiments. Statistical significance was established using an unpaired Student’s T-Test, applying a Bonferroni correction to account for the number of multiple comparisons within each treatment. *p < 0.008; **p < 0.004. (**b**) Changes in total SUMO CNest per 100 ng of RNA triggered by the different stresses used. (**c**) Heat map representation of the data presented in A. Fc: fold change. (**d**). Immunoblot data showing changes in global SUMOylation levels triggered by Influenza A Virus infection, heat-shock, and cold-shock. The membranes were immunoblotted for GAPDH, washed, immunoblotted for SUMO1, stripped using a heat denaturation method, and subsequently blotted for SUMO2/3. 1, Mock infected cells; 2, IAV infected cells; 3, heat-shock control; 4, heat-shock sample; 5, cold-shock control; 6, cold-shock sample. *GAPDH.

In terms of overall changes in total SUMO transcript abundance, out of the three types of stress tested, cold-shock was the only one that resulted in either no changes or a slight increase in total SUMO transcripts. Heat-shock consistently resulted in minor decreases in the abundance of total SUMO transcripts, whereas IAV infection triggered different effects on a cell-dependent manner, causing a doubling in SUMO transcripts in A549 cells and a slight decrease in HEK293A cells (Fig. 4b).

At the level of individual transcript variants, IAV infection consistently increased the abundance of SUMO1V1 and decreased that of SUMO1V2 and SUMO1V3 in both cell lines tested. However, IAV infection triggered increases in all other SUMO variants in A549 cells but decreased them in HEK293A cells. Cold-shock increased the abundance of all S1 variants in both A549 and HEK293A cells but triggered only a small increase in SUMO3V1 in A549 cells and resulted in decreases in SUMO3V1 and SUMO3V2 in HEK293A cells. The SUMO2 variants (SUMO2V1 and SUMO2V2) were not substantially affected by cold shock in either A549 or HEK293A cells. Finally, heat shock resulted in minor changes (less than twofold) below the threshold for statistical significance across all SUMO variants in both A549 and HEK293A cells (Fig. 4a,c).

Immunoblot analyses revealed consistent increases in SUMO1 and SUMO2 SUMOylation triggered by the various stress conditions, as evidenced by increases in SUMO signal in the high molecular weight region of the gel including the stacking. The sole exception to this was cold-shock, which triggered increased SUMO1 and SUMO2/3 SUMOylation in HEK293A cells but failed to do so in A549 cells. To confirm this unexpected result, three independent cold-shock experiments were performed, all producing identical results (Supplementary Fig. S3).

Overall, exposure to most types of stress triggered clear increases in global cellular SUMOylation, as determined by immunoblotting. However, such increases were not accompanied by consistent increases in the abundance of the transcript variants coding for the prototypical SUMO modifiers nor in consistent decreases in the abundance of the transcripts coding for the SUMO alpha isoforms. Instead, the changes observed in transcript abundance were more nuanced and stress-type and cell-type specific.

### The nucleo-cytoplasmic distribution of the SUMO variants is differentially affected by cold-shock

Given that translation is a cytosolic event, mature transcripts must be exported out of the nucleus to allow their efficient use as templates for translation. To determine whether the nuclear export of the different SUMO variants was differentially regulated, we measured the nucleocytoplasmic distribution of the variants in A549 and HEK293A cells. To this end, we performed standard nuclear-cytoplasmic fractionations, purified RNA from each fraction, and measured the CNest for each variant with our validated RT-qPCR approach. Furthermore, to determine whether the nuclear export of the SUMO variants was affected by stress, we also assessed their nucleocytoplasmic distribution after cold-shock. We chose this stress condition because it triggered the smallest changes in SUMO2 splicing processing in both HEK293A and A549 cells, and it triggered a noticeable increase in SUMO2 SUMOylation in HEK293A cells but not in A549 cells as evidenced by immunoblotting. Hence, cold-shock was the type of stress most likely to exert its effects via other post-transcriptional regulatory events. As controls, we assessed the distribution of both, the spliceosomal U2 small nuclear RNA (snRNA), and the ribosomal protein S14 mRNA, two transcripts exhibiting mostly nuclear and cytoplasmic distributions, respectively. In all experiments performed with both A549 and HEK293A cells, more than 74% of U2 was detected in the nucleus while more than 85% of S14 was found in the cytoplasm, therefore demonstrating the validity of the nucleocytoplasmic fractionations performed (Supplementary Fig. S4).

**Figure 5:**
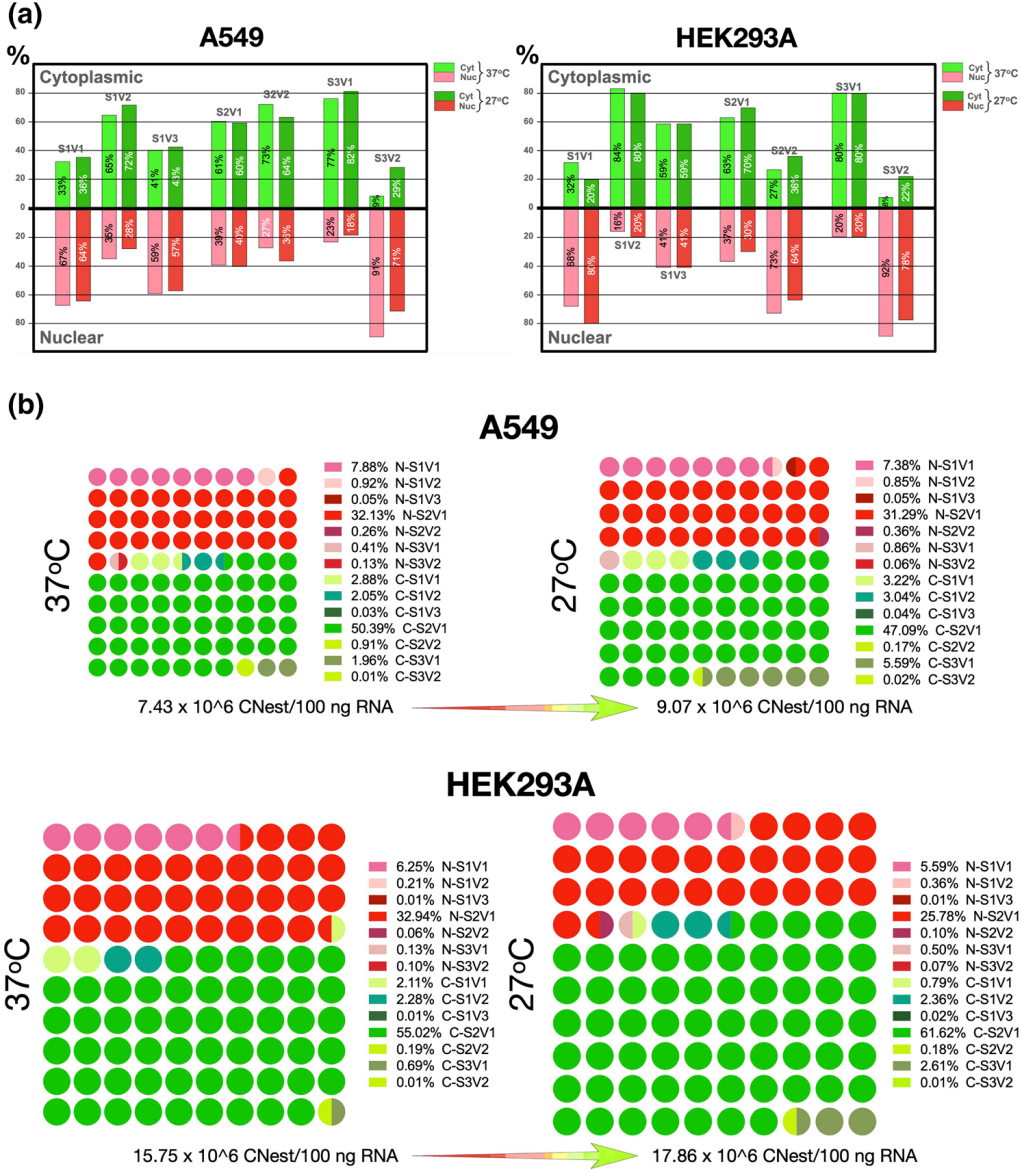
The nucleocytoplasmic distribution of the SUMO variants is differentially regulated upon cold-shock. Cells maintained for 24 h at either 37 °C or 27 °C (cold-shocked) were collected, lysed, and separated into nuclear and cytoplasmic fractions. Total RNA was purified from each fraction and used to estimate nuclear and cytoplasmic CNest for each variant. The data presented correspond to the average values from triplicate measurements obtained in three independent experiments. (**a**) Nucleocytoplasmic distribution of each variant under normalcy (37 °C) and cold-shock (27 °C). In each case, the sum of the nuclear and cytoplasmic fractions equals 100% of the total transcript present in the cell for that variant. (**b**) Dot matrices (10 × 10 dots, 100 dots per matrix) representing the nucleocytoplasmic distribution of each variant as a fraction of the total amount of SUMO transcripts present in the cell under normalcy (37 °C) and cold-shock (27 °C). The total SUMO transcripts were calculated by adding the CNests obtained for the nuclear and cytoplasmic fractions for each variant. The respective abundance of each variant, indicated as a percentage of the total, was calculated and is represented as dots. Each dot represents 1% of the total SUMO transcripts in the cell. The area of the dot matrix is proportional to the total amount of SUMO transcripts present; thus, the total CNest for SUMO transcripts under cold-shock for HEK293A cells is the largest of all, whereas the one for A549 cells under normalcy is the smallest of all. The nuclear fractions are shown in shades of red, while the cytoplasmic fractions are shown in shades of green. The actual percentage corresponding to each variant is shown with their respective color code. The calculated average CNest values for all SUMO transcripts per 100 ng of total RNA under normalcy and cold-shock for each cell line are shown under each matrix. The growing-colored arrows indicate the increase in copy number and cytoplasmic distribution observed upon cold-shock. The data presented represents the average of three independent experiments each measured in triplicate. For the corresponding data presented as bar graphs with error lines, see Supplementary Fig. S5.

Analysis of the nucleocytoplasmic distribution of the SUMO variants indicated differential nuclear retention, with some variants exhibiting a marked predominant nuclear distribution (for instance, SUMO1V1, SUMO1V3, and SUMO3V2), and some exhibiting a marked predominant cytosolic distribution (for instance, SUMO1V2, SUMO2V2, and SUMO3V1). No major differences in the distribution of the SUMO transcripts were observed between A549 and HEK293A cells, with the sole exception of SUMO2V2, which was mostly cytosolic in A549 cells (73% cytosolic) and mostly nuclear in HEK293A cells (73% nuclear). Notably, cold-shock did not trigger significant changes in the nucleocytoplasmic distribution of most variants, with three exceptions: SUMO1V1, whose cytoplasmic distribution decreased by 12%, but only in HEK293A (it increased slightly in A549 cells); SUMO3V2, whose cytoplasmic distribution increased upon cold-shock by more than 20% and 14% in A549 and HEK293A cells, respectively; and SUMO2V2, whose cytosolic distribution decreased by almost 10% in A549 cells but increased by a similar amount in HEK293A cells (Fig. 5a). These findings indicated a differential, cell-specific and variant-specific, nuclear export/retention of the SUMO variants, and a similarly nuanced regulation of their nucleocytoplasmic localization upon cold-shock.

To obtain a more detailed understanding of the potential contribution of the nuclear export/retention of the different SUMO variants toward the regulation of the activity of the SUMOylation system, for each cell type we calculated the total SUMO CNest both at 37 °C and under cold-shock, and then calculated the corresponding fraction contributed by the nuclear and cytosolic fraction of each variant. To facilitate visualization of the data, we chose to represent each set of values obtained using a dot matrix made of a 10 × 10 dot array in which every dot represents 1% of the total of all SUMO transcripts present in the cell (Fig. 5b).

In both, A549 and HEK293A cells, cold-shock triggered increases in the total pool of SUMO transcripts accompanied by increases in the overall cytoplasmic abundance of such transcripts, with the increase in cytoplasmic distribution being substantially larger in HEK293A cells. However, these overall increases in cytoplasmic distribution were dictated by specific variants and did not correspond to consistent increases across all variants, with some variants becoming more nuclear upon cold shock. In A549 cells, the increase in cytoplasmic SUMO transcripts was driven by increases in cytoplasmic SUMO1V1, SUMO1V2, and SUMO3V1, with SUMO3V1 being the most increased (~ 3.6% increase). In HEK293A cells, the increase in cytoplasmic SUMO transcripts was driven by increases in cytoplasmic SUMO1V2, SUMO2V1, and SUMO3V1, with SUMO2V1 being the most increased (~ 6.6% increase). In contrast, the transcripts that displayed the largest decreases in cytoplasmic abundance were SUMO2V1 in A549 cells (~ 3.3% decrease), and SUMO1V1 in HEK293A cells (~ 1.32%). The decreases in cytoplasmic abundance upon cold-shock for these transcripts were in part due to decreases in their overall abundance. In A549 cells, SUMO2V1 went from representing 82.5% to representing only 78.4% of all SUMO transcripts; in HEK293A cells, SUMO1V1 went from representing 8.4% to representing only 6.4% of all SUMO transcripts (Fig. 5b and Supplementary Fig. S5).

Importantly, the increase in cytoplasmic SUMO2V1 in HEK293A upon cold-shock did not correlate with a net increase in the amount of the SUMO2V1 transcript, as this transcript represented about 87% of all SUMO transcripts in both normalcy and cold-shock. This indicates that the nuclear export of SUMO2V1 is substantially increased upon cold-shock in HEK293A cells. In contrast, both the total amounts and the cytosolic percentage of SUMO2V1 were decreased upon cold-shock in A549 cells. Considering that SUMO2/3 SUMOylation was clearly increased by immunoblot in HEK293A cells but not in A549 cells, the regulation of the nuclear export of the SUMO transcripts appears to be an important contributing factor toward the global regulation of cellular SUMOylation upon cold-shock.

### The SUMO alpha isoforms are likely to be translated and expressed in the cell, albeit at low levels

The cytoplasmic localization of a given transcript is a strong indicator of its potential functionality as a template for translation, as translation is a cytoplasmic event. Thus, the demonstration of the existence of cytoplasmic forms of the variants coding for the SUMO alpha isoforms (i.e., SUMO1V3, SUMO2V2, and SUMO3V2) indicated that the SUMO alphas were likely to be translated and could therefore be present in the cellular environment. To determine with more certainty whether the SUMO alpha protein isoforms are produced in the cell, we searched for direct proof by mining Ribo-seq data. To this end, we chose five different Ribo-seq studies at random among those currently available in the NCBI databases and then searched for select sequence strings corresponding to the nucleotide sequences spanning between 26 and 30 nucleotides around exon-exon junctions specific for SUMO1V3, SUMO2V2, and SUMO3V2, using the SeqKit tool as described in “Methods”.

**Figure 6:**
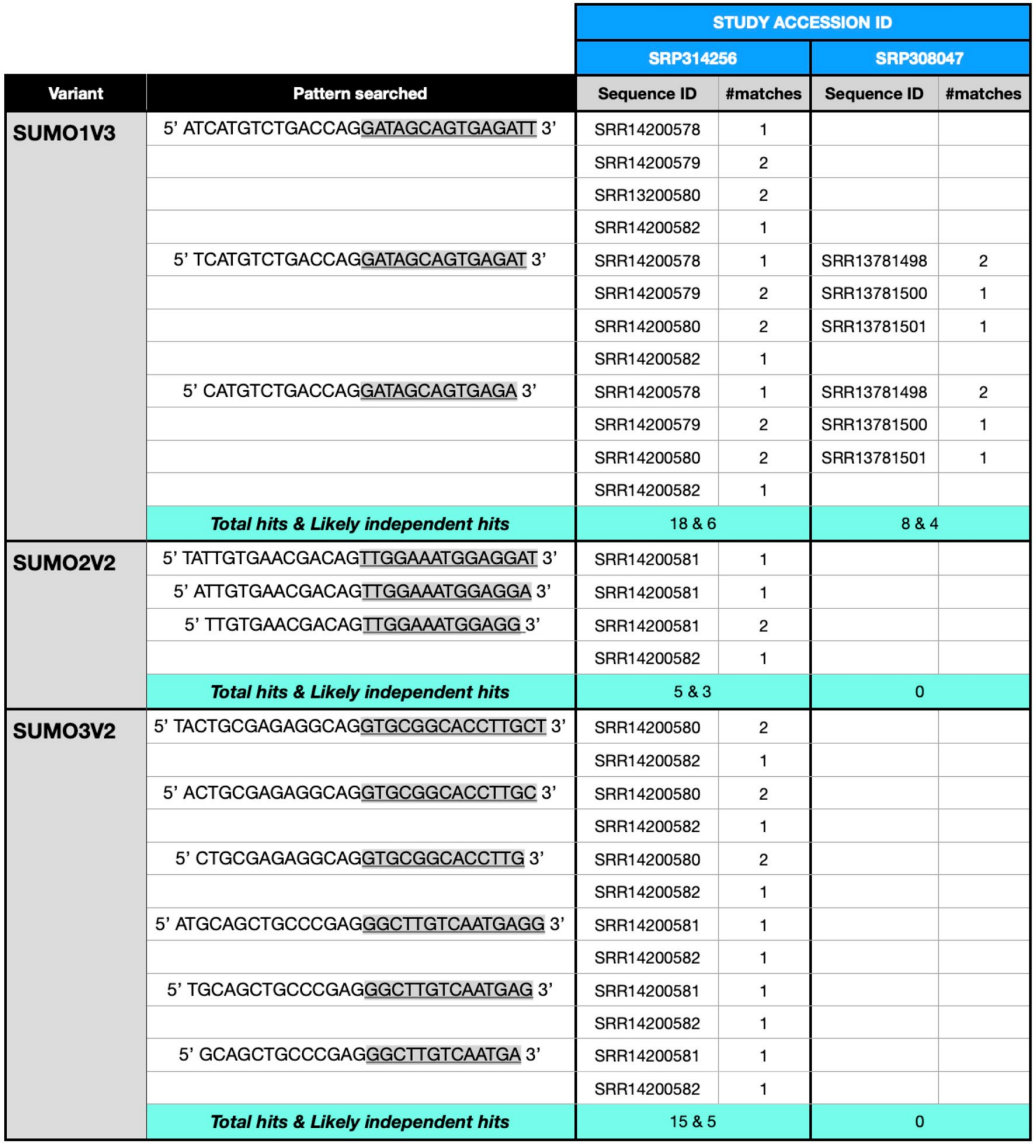
Ribo-seq data provides evidence that the transcripts coding for the SUMO alpha isoforms are translated. Five different Ribo-seq studies chosen at random from the NCBI databases were mined to search for the sequences corresponding to exon-exon junctions specific for the SUMO1V3, SUMO2V2, and SUMO3V2 transcripts shown under “Pattern searched.” For each sequence, the nucleotides presented with clear background correspond to the upstream exon, whereas the underlined nucleotides shown with a gray background correspond to the downstream exon. The Sequence IDs for the datasets containing hits, as well as the number of hits on each sequence dataset, are shown, as are the total number of hits found and the likely independent hits. Three out of the studies mined gave no hits and correspond to study accession IDs SRP122522, SRP362491, and SRP286677. Notice however that shortening the sequences used to mine the datasets by two additional nucleotides (one at the 5’ end and one at the 3’ end) resulted in an increase in the number of hits and all studies giving at least one hit, with the sole exception of study accession ID SRP362491, which still gave no hits.

These analyses confirmed that the three variants coding for SUMO alpha isoforms, i.e., SUMO1V3, SUMO2V2, and SUMO3V2, are in fact found in translating ribosomes. For SUMO1V3, we found 10 independent hits distributed among two out of the five different datasets analyzed. For SUMO2V2, we found 3 independent hits in one of the five datasets analyzed. Finally, for SUMO3V2, we found 5 independent hits in one of the five datasets analyzed (Fig. 6). These findings provided conclusive evidence that the variants coding for the SUMO alpha isoforms are translated and therefore the SUMO alpha proteins are likely to be present within human cells.

### The SUMO alphas exhibit patterns of cellular localization clearly different from that of their prototypical SUMO counterparts

Having confirmed that the SUMO alphas are translated in human cells, we aimed to assess the functional properties of the SUMO alphas. To this end, we compared the predominant cellular localization of the SUMO alphas with that of their respective prototypical SUMO proteins. This was achieved by implementing a transfection approach with plasmids coding for N-terminal YFP-fusions of the prototypical SUMO proteins and their respective SUMO alphas, ending in the di-glycine motif. Thus, the YFP-SUMO fusions produced correspond to mature (proteolytically processed) SUMO molecules, ready for conjugation.

**Figure 7:**
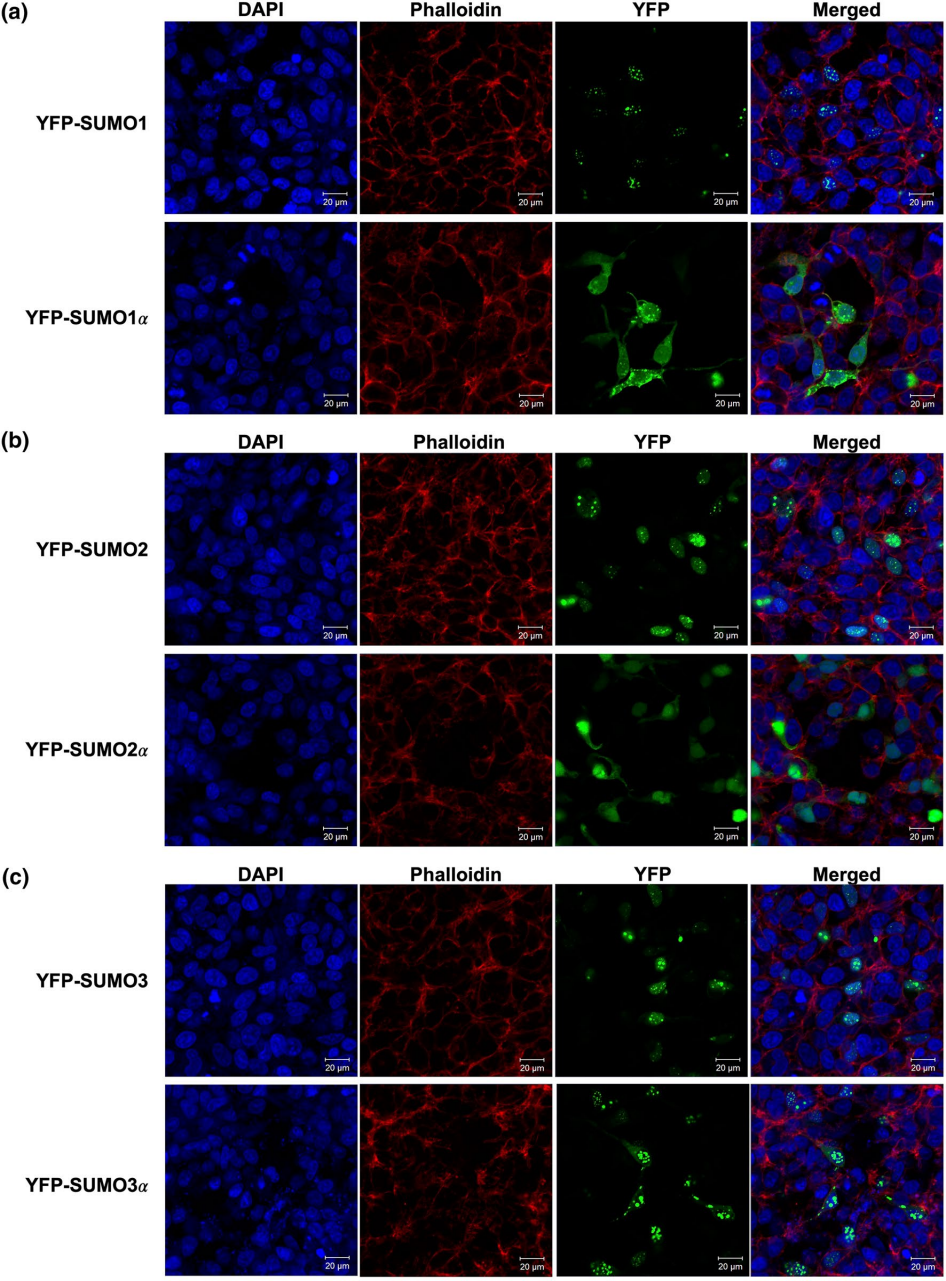
The SUMO alphas exhibit different cellular localization patterns from those associated with their prototypical isoform. HEK293A cells were transfected with expression constructs coding for YFP-fusion forms of either the prototypical SUMO proteins or their associated SUMO alpha isoforms. At 24 h post-transfection, the cells were fixed, permeabilized, treated with Phalloidin (a bicyclic peptide that stains all types of actin filaments) and DAPI (a DNA-binding fluorescent dye), and images were captured by confocal microscopy. The images provided constitute representative fields. (**a**) YFP-SUMO1 and YFP- SUMO1α. (**b**) YFP-SUMO2 and YFP- SUMO2α. (**c**) YFP-SUMO3 and YFP- SUMO3α. The fluorescence associated to each marker and its respective color representation are as follows: DAPI, blue; Phalloidin, red; YFP, green.

YFP-SUMO1 appeared to be distributed exclusively in well-defined dots contained within the nucleus, present at around 8–16 dots per nucleus. In contrast, YFP-SUMO1α exhibited diffuse cytosolic and diffuse nucleoplasmic localizations and appeared to also be present in dot structures present in both the nucleus and the cytoplasm but that appeared more abundant in the cytoplasm (Fig. 7a).

YFP-SUMO2 showed exclusive nuclear localization and appeared to be distributed both, in dot structures present at 3–11 dots per nucleus, and in a diffuse pattern equally distributed across the nucleus. In contrast, YFP-SUMO2α displayed a predominantly nuclear profile, being present as a diffuse pattern equally distributed across the nucleus, but also exhibited a diffuse homogeneous distribution throughout the cytoplasm (Fig. 7b).

YFP-SUMO3 showed a similar distribution to that exhibited by YFP-SUMO2, displaying an exclusive nuclear distribution characterized by the presence of dot structures present at 1–14 dots per nucleus, and a diffuse nucleoplasmic pattern. In contrast, YFP-SUMO3α displayed both, the presence of nuclear dot structures at 3–16 dots per nucleus, and a diffuse cytoplasmic pattern equally distributed throughout the cytoplasm, while lacking any diffuse nuclear fluorescence (Fig. 7C).

Immunoblot analyses of cells transfected with the plasmids coding for the N-terminal YFP-fusions showed the absence of truncated forms for the YFP-fusion proteins produced (Supplementary Fig. S6). Therefore, the cellular distribution patterns for the different YFP-SUMO proteins described above reflect those of their SUMO components.

Altogether, the localization of the prototypical SUMO proteins, i.e., SUMO1, SUMO2, and SUMO3, was consistent with previously reported data by various groups, while the localization of the SUMO alpha proteins, i.e., SUMO1α, SUMO2α, and SUMO3α, appeared clearly different from that of their prototypical counterparts. These differences indicated that the SUMO alphas were likely to be functionally different from the prototypical SUMOs.

### SUMO3α is the only SUMO alpha that appears to be conjugatable

Next, we evaluated the predicted structures of the SUMO alphas for likely functional effects. To this end, we performed Alpha Fold and RaptorX structure predictions of the SUMO alphas and looked for disruptions in known functional motifs and structures present in the prototypical SUMO proteins.

**Figure 8:**
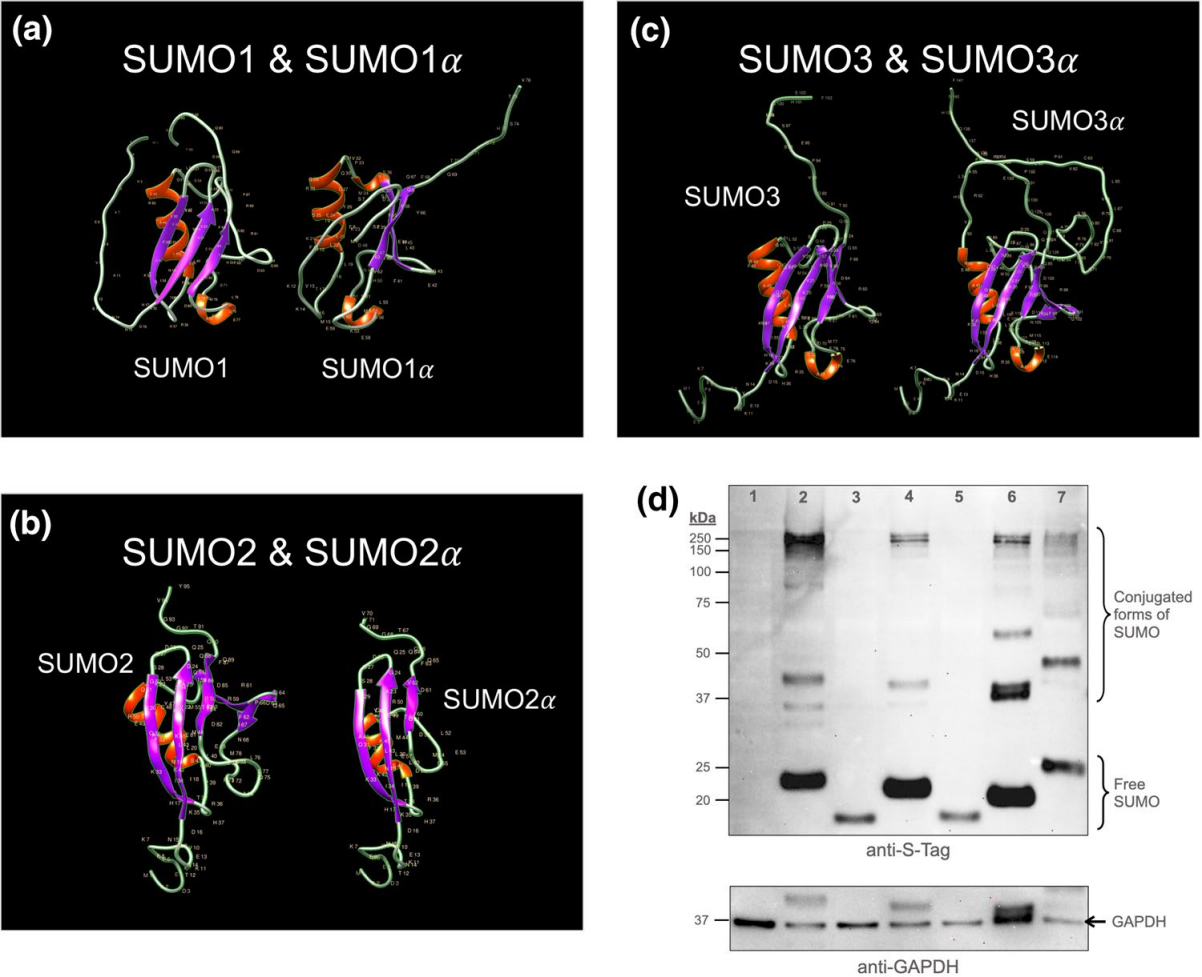
The exon-deficient SUMO alphas (SUMO1α and SUMO2α) are non-conjugatable. (**a**–**c**). Crystal structure of the SUMO alphas as compared to those of their prototypical isoform as predicted by RaptorX. The images shown were considered to best emphasize the differences in predicted crystal structures. Notice how the additional sequence present in SUMO3α is predicted to form an unstructured region that remains outside the central globular core of SUMO3α, therefore imposing minimal disruptions in the globular core; in sharp contrast, the specific amino acid sequence deletions present in SUMO1α and SUMO2α are predicted to trigger substantial changes in their globular core as compared with their prototypical isoforms. Virtually identical tertiary structures were predicted by Alpha Fold analyses. (**d**) Immunoblot analyses showing the patterns of conjugation associated to each SUMO isoform. HEK293A cells were transfected with expression constructs coding for the indicated His-S-tagged SUMO protein. At 24 h post-transfection, the cells were lysed and the resulting extracts analyzed by SDS-PAGE and immunoblotting. Upper panel: immunoblot performed with antibodies directed against the S-tag (located near the N-terminal end of the proteins). Lower panel: subsequent immunoblot using anti-GAPDH antibodies as loading control for the different samples. The localization of free- and conjugated-SUMO forms is indicated. High molecular weight signals are easily visible for SUMO3α, indicating its ability to become conjugated to other proteins. In contrast, no high molecular weight forms were observed for SUMO1α and SUMO2α. 1, Mock transfected cells; 2 and 3, cells over-expressing SUMO1 and SUMO1α, respectively; 4 and 5, cells over-expressing SUMO2 and SUMO2α, respectively; 6 and 7, cells over-expressing SUMO3 and SUMO3α, respectively. All over-expressed SUMO proteins contained tandem His- and S-tags at their N-terminus.

No differences were observed between the structures predicted by the Alpha Fold and the RaptorX analyses. Both analyses predicted that SUMO1α and SUMO2α contained substantial alterations in the characteristic β-grasp fold structure of their prototypical isoforms. Given the nature of such alterations, they were predicted to disrupt SUMO1α and SUMO2α’s ability to interact with the enzymatic components of the SUMOylation system and make them non-conjugatable (Fig. 8a,b). For SUMO3α, the models predicted that the extra 38 amino acid residues added by the alternative splicing event formed a long unstructured flexible loop that remained away from the β-grasp fold structure, without affecting any critical surface on SUMO3 (Fig. 8c). Thus, SUMO3α was predicted to be conjugatable.

To empirically test the conjugatability of the SUMO alphas we used a transfection approach using plasmid constructs coding for N-terminally His-S-tagged SUMO proteins. The plasmids were transfected into HEK293A cells and, 24 h post-transfection, the cells were collected, and the resulting cell extracts analyzed by immunoblotting using anti-S tag antibodies.

As expected, all three prototypical SUMO proteins, i.e., SUMO1, SUMO2, and SUMO3, produced high molecular weight signals readily visible by immunoblotting, indicative of their ability to become conjugated to a large array of proteins; additionally, all three were also readily detected in their unconjugated forms at their expected molecular weights. In contrast, out of the three SUMO alpha isoforms, only SUMO3α produced high molecular weight forms, although their profile appeared different from that observed for SUMO3. SUMO1α and SUMO2α did not produce detectable high molecular weight forms, even in over-exposed images, and their free unconjugated forms, while consistent with their expected molecular weight, exhibited substantially decreased intensity, suggesting that SUMO1α and SUMO2α were probably unstable (Fig. 8d). Thus, SUMO3α was the only conjugatable alpha isoform, although the pool of proteins targeted for conjugation with SUMO3α was probably different from that conjugated with SUMO3.

To confirm the data indicated above and determine whether SUMO1α and SUMO2α were targeted for proteasomal degradation, we repeated the experiment above but treated the cells with MG132 for the last 4 h prior to sample collection. Treatment with MG132 resulted in increased signals for SUMO1α and SUMO2α, thus demonstrating that these proteins are more unstable than their prototypical counterparts and that their degradation is proteasomal-dependent. However, no high-molecular weight signals were observed for SUMO1α and SUMO2α despite their increased detection, thus confirming that they are not conjugatable. MG132 treatment also increased the signal of all SUMOs, thus supporting proteasomal degradation as part of the regulatory mechanisms that control SUMO levels in the cell (data not shown).

Altogether, these analyses demonstrated that the SUMO alphas were functionally different from their prototypical counterparts. SUMO3α was the only SUMO alpha that proved to be conjugatable to cellular targets in vivo, although it appeared to exhibit differential targeting from that of SUMO3. SUMO1α and SUMO2α were not conjugatable and exhibited decreased stability.

## Discussion

The five SUMO paralogs expressed in humans, encoded by five different genes, are frequently referred to as “SUMO isoforms” in the literature. Such use of the term “isoforms” is incorrect, as isoforms are proteins encoded by the same gene that differ in their primary structure because of alternative splicing events or alternative translational start sites that alter the coding sequence of their transcripts^59^. Thus, while the different mature mRNA transcripts derived from the SUMO genes that were analyzed in this study were deposited in the NCBI database several years ago, the existence of actual protein isoforms for the main human SUMO paralogs had not been previously reported. Therefore, this is the first report addressing the existence and functional characterization of protein isoforms for the main human SUMO proteins, SUMO1, SUMO2, and SUMO3. Our data strongly supports that such SUMO isoforms, which we have named SUMO1α, SUMO2α, and SUMO3α, are translated and therefore are likely to contribute to the overall pool of SUMO proteins in the cell. Our findings also indicate that the SUMO isoforms differ from their prototypical counterparts not only in sequence and structure but also in cellular localization and function.

The abundant RNA-seq data deposited in the NCBI database during the last quindecennium allowed the identification of the different variant mRNA transcripts reported here. As the number of RNA-seq studies continues to increase almost weekly, so does the pool of mature transcripts deposited in databases. Thus, the variants described and characterized in this study do not intend to represent the totality of all SUMO transcripts. While the number of validated variants for the SUMO2 and SUMO3 paralogs has remained unchanged at two variants each, at the time these studies were started there were only three validated mature mRNA variants for the SUMO1 gene. Four new transcript variants for the SUMO1 gene have been added to the NCBI database since then; of those, two code for additional SUMO1 isoforms. The first corresponds to a transcript lacking exon 4, thus coding for a shorter isoform. The second corresponds to a transcript containing an additional exon between exon 4 and exon 5, thus producing a larger SUMO1 isoform carrying 45 additional amino acid residues near the C-end. These new SUMO1 variants add further complexity to the potential regulatory role played by alternative splicing on the overall control of cellular SUMOylation. However, given that the new variants were reported only recently, it is likely that their overall abundance is substantially lower than that of the variants characterized in this report and, therefore, those newly identified variants may contribute minimally to the overall control of SUMO1 expression. Likewise, additional variants that may be found in future studies are likely to correspond to mature transcripts produced either in much fewer quantities than the ones we addressed here, or only in a limited type of cells under very specific conditions. Therefore, while the variants we presented in this report do not constitute the totality of all SUMO transcripts in human cells, they are likely to constitute the best represented and the primary contributors to the total pool of SUMO transcripts in most human cells.

To assess the contribution of alternative splicing toward the regulation of global cellular SUMOylation, we first performed an exhaustive evaluation of the levels of each transcript under normal conditions in four different cell types. Three of the cell types analyzed were well-characterized cell lines exhibiting hypotriploid chromosomal numbers, thus PBMCs were included in our analyses to provide some degree of comparison with a population of normal cells. All cell types analyzed demonstrated to have a marked predominance of SUMO2V1 transcripts, ranging from 63% of the total SUMO transcripts (in PBMCs) up to 90% in HEK293A cells. Importantly, in every cell type analyzed SUMO2V1 constituted almost the totality of the mature mRNA for SUMO2, with SUMO2V2 constituting at most 0.73% of the total SUMO2 transcripts (in A549 cells). Therefore, unlike SUMO1 and SUMO3, for which alternatively spliced transcripts add up to more than 12% of the total cellular transcripts, for SUMO2 the total amount of transcripts appears almost equivalent to the amount assessed for its normally spliced transcript, SUMO2V1. Considering this, and extrapolating it with previously published data^9,49^, SUMO2V1 is likely to constitute the most abundant SUMO transcript in most adult human organs, representing in average about 45% of all SUMO transcripts, and supporting a critical role for SUMO2 in normal adult tissues.

Intriguingly, our data suggest that SUMO2 transcripts are even more abundant in tumor-derived cell lines than in normal adult tissues. This observation, supported by other studies both at the transcript^9^ and protein^49^ levels, raises the question of whether tumor development and progression promotes enhanced SUMO2 expression, whether increased SUMO2 expression promotes tumor development and progression, or whether SUMO2 expression and tumor progression are part of a positive feedback loop in which both components promote each other. While future studies aimed at answering this question are likely to provide interesting insights into SUMO function and regulation, the predominance of SUMO2 in tumor cells makes it the ideal SUMO paralog target for anti-tumor therapeutics.

The analyses we present in this study indicate that none of the three stressors that we chose (namely, IAV infection, cold-shock, and heat-shock) consistently increased all the transcripts coding for the prototypical SUMO isoforms while simultaneously decreasing the transcripts coding for the SUMO alpha isoforms. Instead, the changes observed in the abundance of the different SUMO variants appeared to be stress-type and cell-type specific. Interestingly, some of the stress-induced changes were relatively large, exceeding a twofold increase, which indicate that they could potentially account for most of the increases in global SUMOylation observed. For example, in A549 cells IAV infection triggered a ~ twofold increase in SUMO1V1, SUMO2V1, and SUMO3V1, thus accounting for the approximate doubling in SUMO1 and SUMO2/3 SUMOylation observed in those cells. Similarly, in HEK293A cells IAV infection triggered a ~ twofold increase in SUMO1V1 levels but not in SUMO2V1 or SUMO3V1; this matched closely the apparent increases in SUMO1 and SUMO2/3 SUMOylation observed upon IAV infection in HEK293A cells. Therefore, there appears to exist a close correlation between transcript variant abundance and overall SUMOylation levels during IAV infection. This close correlation was not true for the other types of stress. Importantly, the SUMOylation increases triggered by IAV infection are only visible after about 9 h post-infection, which provides the time needed for an increase heavily dependent on transcription and transcript processing.

Heat shock triggered the largest apparent increases in global cellular SUMOylation observed by immunoblotting in both A549 and HEK293A cells. However, at the transcript level heat shock did not trigger significant increases in the abundance of any SUMO transcript in the two cell lines tested. It is therefore possible that the net increase in SUMO modifiers likely needed to allow the large increase in global cellular SUMO1- and SUMO2/3-SUMOylation triggered by heat-shock might depend upon other mechanisms. Likely candidates include regulation of nucleocytoplasmic traffic, which seems to play an important role in cold-shock-induced SUMOylation (see below), and translational regulation, which was not evaluated in this study but would fit better the short time required for the increases observed, which become visible after only 30 min.

Cold-shock increased all SUMO1 variants in both A549 and HEK293A cells. However, A549A cells did not display any apparent cold-shock-triggered increase in global SUMOylation, neither for SUMO1 nor for SUMO2/3. HEK293A cells did display a noticeable cold-shock-induced increase in SUMO1 and SUMO2/3 SUMOylation, but the SUMO2/3 increase was not accompanied by substantial increases in SUMO2V1 or SUMO3V1 abundance. Instead, the increase in SUMO2/3 SUMOylation observed in HEK293A cells appeared to correlate with an increase in the nuclear export of the SUMO2V1 transcript, which went from being 55% cytoplasmic to being 61% cytoplasmic upon cold-shock. This indicates that the regulation of nucleocytoplasmic export of the SUMO transcripts is a critical regulatory point for the cold-shock-induced increase in global cellular SUMOylation. Interestingly, our analyses showed that the nuclear retention of one specific transcript, SUMO3V2, is consistently increased upon cold-shock in both cell lines analyzed. SUMO3V2 is the most abundant variant coding for a SUMO alpha isoform, and its protein product, SUMO3α, is the only conjugatable SUMO alpha isoform. This data suggests that SUMO3α could play an antagonistic role thus imposing a need to prevent its expression to allow increases in global SUMOylation.

One critical consequence of alternative splicing is the production of protein isoforms exhibiting different functional properties from those displayed by the prototypical protein encoded by a gene. However, for this to be possible, the alternatively spliced transcripts must be exported to the cytoplasm and translated by ribosomes. Our data indicate that all the variants coding for the SUMO alpha isoforms are exported to the cytoplasm, albeit with different efficiencies, and are actively translated by ribosomes, as supported by the finding of sequences specific for such variants among the pools of Ribo-seq data analyzed. This supports the likelihood that the SUMO alpha isoforms are in fact present in the cell and may therefore provide added regulatory functionality to the SUMOylation system.

While the Ribo-seq data strongly supports the existence of the SUMO alphas in the cell, mass spectrometry data identifying peptides exclusive of the SUMO alphas would provide unquestionable evidence for the existence of the SUMO alpha isoforms in the cellular milieu. We attempted to detect such tryptic peptides in data sets generated during normal proteomic screenings; however, our attempts proved unsuccessful. We consider that the failure to achieve such evidence is due to four factors: first, there are limited tryptic fragments that are exclusive to the SUMO alphas, i.e., tryptic fragments that are not present in their corresponding prototypical proteins. Specifically, for both SUMO1α and SUMO2α there is only one exclusive tryptic peptide, and for SUMO3α there are two. Second, all the exclusive peptides are longer than 12 amino acid residues (Supplementary Table S2), which tend to be slightly less represented than shorter peptides in tryptic proteomic data pools. Third, the prototypical SUMO proteins themselves usually exhibit relatively poor coverage in normal proteomic screenings, i.e., a few tryptic cleavage products are rarely seen, and overall coverage rarely exceeds 60%. Lastly, the SUMO alpha proteins, being encoded by mRNAs that constitute less than a twentieth of the mRNA coding for their corresponding prototypical SUMOs, are likely to be present at very low cellular concentrations. We are currently attempting the development of peptide-specific antibodies that might allow us to specifically detect the SUMO alphas by immunochemical approaches to pursue further functional studies.

Our immunoblot data obtained using over-expressed tagged SUMO alphas indicated that SUMO3α is conjugatable but SUMO1α and SUMO2α are not. This agrees with the structural models predicted by our Alpha Fold and RaptorX analyses, and by structural analyses of the prototypical SUMOs in interaction with the enzymatic players of the SUMOylation cascade. Interestingly, the non-conjugatable SUMO alphas (SUMO1α and SUMO2α) exhibited a more dissimilar cellular localization from that of their respective prototypical SUMOs than the only conjugatable SUMO alpha, SUMO3α. The main changes in cellular distribution observed for the SUMO alphas were a substantial decrease in the ability to form large dense SUMO complexes/speckles and the occurrence of a diffuse cytosolic distribution not visible in the prototypical SUMOs. Considering that SUMOylation is now recognized as a mediator of some of the liquid–liquid phase separation events that result in the formation of membrane-less organelles^60^, it is possible that the non-conjugatable SUMO alphas may lack the ability to drive liquid–liquid phase separation events, thus explaining their decreased association to speckles and increased diffuse distribution.

The process of SUMO activation and conjugation requires specific protein–protein interactions that are established between the enzymatic components of the SUMOylation system and the SUMO modifiers. Those interactions are mediated by specific amino acid residues in the SUMO modifiers and the activating and conjugating enzymes.

For the activation stage, there are numerous well-characterized residues in the SUMO modifiers that are involved in making contacts with the SAE2 component of the E1 conjugating enzyme (the SAE1 component doesn’t establish direct interactions with the SUMO modifiers). Such residues include Gln29, Ser31, Asn60, Arg70, Glu89, Tyr91, Glu93, Gln94, Thr95, Gly96, and Gly97 in SUMO1, and Gln25, Gly27, Arg56, Pro66, Asp85, Phe87, Gln89, Gln90, Thr91, Gly92, and Gly93 in SUMO2^61^. Out of those, Gln29 is absent in SUMO1α while Arg56 and Pro66 are absent in SUMO2α. The absence of such amino acid residues is likely to prevent SUMO1α and SUMO2α from forming functional interactions with SAE2, thus precluding their normal activation.

For the conjugation stage, the SUMO modifiers establish two different types of interactions with the Ubc9 (E2) conjugating enzyme. The first, driven by the E1-SUMO complex, which mediates the transference of SUMO from the E1 to the E2 enzyme, appears dependent on residues Gln29, Arg63, Gln92, Gln94, Thr95, Gly96, and Gly97 in SUMO1, and residues Gln25, Arg59, Gln88, Gln90, Thr91, Gly92, and Gly93 in SUMO2. The second constitutes a non-covalent interaction that appears important for SUMO chain formation, and is mediated by residues Gln29, Glu33, Arg63, Leu65, Glu67, Gly81, Glu85, Asp86, Val87, Glu89, and Tyr91 in SUMO1, and Gln25, Val29, Arg59, Arg61, Asp63, Glu77, Glu81, Asp82, Thr83, Asp85, and Phe87 in SUMO2^62–65^. Out of all the residues indicated to mediate some type of interaction with Ubc9, Gln29 is absent in SUMO1α while Arg59, Arg61, and Asp63 are absent in SUMO2α. The lack of those amino acid residues is likely to render SUMO1α and SUMO2α unable to interact with Ubc9, therefore preventing them from being conjugatable.

Notice that the absence of a single amino acid residue, Gln29, is likely responsible for SUMO1α’s inability to interact with both the activating and the conjugating enzymes. Although Gln29 is known to establish close contacts with both SAE2 and Ubc9, it is possible that in its absence the efficiency of the activation and conjugation steps may decrease substantially but remain achievable. In support of this possibility, in one of the immunoblots we performed while repeating the experiments shown in Fig. 8d, we observed a minor band for SUMO1α in the molecular weight range expected for SUMOylated RanGAP. As RanGAP is the main cellular target for SUMO1, and SUMOylated RanGAP is partially protected from deconjugation by the SUMO isopeptidases when in complex with RanBP2 and Ubc9^48^, should SUMO1α be even slightly conjugatable, the most likely target it may be found conjugated to is RanGAP. However, considering that the conjugation of the SUMO alphas to cellular targets was assessed using transfection as a way to ensure over-expression of the SUMO alphas, the likelihood that SUMO1α may become conjugated to RanGAP under normal expression levels is probably very low.

Importantly, even though our data indicates that SUMO1α and SUMO2α are not conjugatable, the possibility remains that these non-conjugatable SUMO isoforms may still be able to interact with the E1 and E2 SUMO enzymes and form complexes that render them inactive, as has been postulated by Zhao et al. for peptides representing C-terminal sequences of the prototypical SUMO modifiers^66^.

In addition to their conjugatability, the SUMO proteins achieve some of their critical regulatory roles in the cell by virtue of their ability to establish non-covalent interactions with innumerable proteins containing so-called SUMO Interacting Motifs (SIMs). The region in SUMO1, SUMO2, and SUMO3 involved in interacting with the classical SIM comprises residues F36-Y51 in SUMO1 and Q30-Y46 in SUMO2 and SUMO3^67^. All of those residues are present in the SUMO alphas and their overall structure does not appear disrupted. Therefore, it is very likely that all SUMO alphas may still be able to interact with proteins containing classical SIMs. Considering that SIMs mediate the formation of protein complexes between SUMOylated proteins and other proteins, and are a likely contributor to the phenomenon known as group SUMOylation^68^, it is possible that the non-conjugatable SUMO alphas (SUMO1α and SUMO2α) may regulate some of the SUMO-dependent events that occur in the cell by interacting with SIM-containing proteins. Such interactions could provide antagonistic and/or synergistic functions. Thus, whether the SIM-binding surfaces in SUMO1α and SUMO2α are functional must be empirically tested.

The stability of the SUMO alphas could greatly affect their functional relevance in the cell. While the His-S-tagged N-terminal fusion proteins we over-expressed by transfection to determine the conjugatability of the SUMO alphas appeared substantially less stable than their His-S-tagged prototypical counterparts, the YFP-SUMO alphas used for cellular localization analyses appeared substantially more stable, exhibiting cellular concentrations that seemed higher than those of their prototypical YFP-SUMOs counterparts. Thus, it will be important to determine the stability of the non-tagged SUMO alphas and assess whether they are processed by the cellular SUMO-peptidases to generate mature proteins. We are currently pursuing an in-depth functional characterization of the SUMO alphas to better understand their potential role in the cell. We are also assessing the effects of altering the proportion at which the different variants are produced, using a splicing-targeting approach. Finally, we are also pursuing the characterization of the splicing events for the mRNAs coding for the E1 and E2 enzymes in the SUMO system. These studies could vastly expand the range of SUMO-targeted therapies in the clinic^69^.

## Conclusion

Alternative splicing greatly expands the coding potential of mammalian genomes. The data we present in this report indicates that alternative splicing also contributes to regulating master regulators of cellular physiology like the SUMOylation system. More importantly, our data also provides evidence that protein isoforms of the prototypical SUMO proteins are produced in the cell. The potential regulatory role played by these SUMO isoforms, which we have dubbed the SUMO alphas, remains to be fully explored. A deeper understanding of the mechanisms governing the activity of the SUMOylation system could greatly facilitate the development of SUMO-based therapies and maximize the therapeutic potential of the SUMOylation system.

## Methods

### Primer design approach

To design primer pairs specific for each transcript variant produced by the *SUMO1*, *SUMO2*, and *SUMO3* genes, we first developed a map relating each gene with its mature mRNA transcript variants based on RNA-seq data from the NCBI database. The NCBI database identifiers for the *SUMO* gene sequences used in the analyses are as follows. *SUMO1*: NC_000002.12 Chromosome 2, reference GRCh38.p14; *SUMO2*: NC_000017.11 Chromosome 17, reference GRCh38.p14; *SUMO3*: NC_000021.9 Chromosome 21, reference GRCh38.p14.

The NCBI database identifiers for the *SUMO1* gene transcripts used are as follows: SUMO1 Variant 1 (**SUMO1V1**): NM_003352.8; SUMO1 Variant 2 (**SUMO1V2**): NM_001005781.2; SUMO1 Variant 3 (**SUMO1V3**): NM_001005782.2.

The NCBI database identifiers for the *SUMO2* gene transcripts used are as follows: SUMO2 Variant 1 (**SUMO2V1**): NM_006937.4; SUMO2 Variant 2 (**SUMO2V2**): NM_001005849.2.

The NCBI database identifiers for the *SUMO3* gene transcripts used are as follows: SUMO3 Variant 1 (**SUMO3V1**): NM_006936.3; SUMO3 Variant 2 (**SUMO3V2**): NM_001286416.2.

For designing transcript variant-specific primer pairs, we focused primarily on exon-exon junctions, placing special emphasis in those that were variant-specific. However, if the distance to the next exon-exon junction was either too short or too long, then attention was also given to intra-exonic sequences, particularly if the exon was variant-specific. The specific criteria used for primer design was as follows: (1) Paired primers should have similar melting temperatures. (2) The expected PCR products produced should be between 150 and 350 bp in length. (3) A given primer pair should amplify only one mature mRNA variant. (4) The base composition of the primers should be as close as possible to 50:50 (GC): (AT), and neither (GC) nor (AT) should exceed 60%. (7) All primers should have a clamping sequence (CG, GC, GG, or CC) at their 3’ end. (8) Primers should be free of sequences likely to form stable secondary structures, single primers should not form stable homodimers, and primer pairs should not form stable heterodimers.

All primers were obtained from IDT (Integrated DNA Technologies, Inc., Coralville, IA), reconstituted in sterile TE at a concentration of 100 μM, and further diluted to 10 μM in TE to be used in RT-PCR and RT-qPCR reactions. The sequences of all primers used in this study are provided in Supplementary Table S1.

### Cell and tissue culture

Peripheral Blood Mononuclear Cells (PBMCs) were a gift from Dr. June Kant-Mitchell; these cells had been collected from healthy volunteers, who had provided written informed consent according to a previously approved protocol at the University of Texas at El Paso (UTEP), and kept frozen as 1 mL aliquots at approximately 1 × 10^6^ cells per mL at − 80 °C, with each vial corresponding to cells from one volunteer only. Approval for the use of the PBMCs was obtained from the Institutional Review Board (IRB) Committee at UTEP as well as from the granting institution, U.S. Army Medical Research and Development Command, Office of Research Protections, Human Research Protection Office. When needed, the PBMCs were thawed and directly used for RNA purification as described below. A total of three different vials, from three different individuals, were used in these studies. Human embryonic kidney cells (HEK293A) were from Invitrogen (ThermoFisher Scientific, Inc., Waltham, MA). A549 and Calu-3 cells were from ATCC (American Type Culture Collection). HEK293A, A549, and Calu-3 cells were grown at 37 °C, 5% CO_2_, in 1 × Complete Medium consisting of 1 × DMEM containing high glucose, pyruvate, and GlutaMAX™ (Gibco™, ThermoFisher Scientific, Inc.), supplemented with 10% Fetal Bovine Serum.

### RNA purification

For RNA purification from PBMCs, one vial of frozen cells was thawed on ice, lysed with 200 μL of buffer RLT, and processed as described below. For RNA purification from A549, Calu-3, or HEK293A cells, cells were plated at 3 × 10^5^ cells per well on a 6 well plate, cultured for 36 h at 37 °C, 5% CO_2_, washed in 1 mL 1 × PBS, and lysed with 200 μL of buffer RLT. Total RNA was purified using the Qiagen RNeasy Mini Kit® via the Qiashredder® method (both from QIAGEN, Inc., Redwood City, CA), as recommended by the manufacturer. The purified RNA was eluted off the column using 50 μL of RNase-free milli-Q water, aliquoted in 9 μL aliquots and stored at -80 ºC.

### Assessment of purified RNA quality and quantity

Purified RNA was quantified using a Qubit Fluorometer 3.0® (ThermoFisher Scientific, Inc.) following the manufacturer’s instructions. To check the quality of the RNA purification, each sample was analyzed using formaldehyde-agarose gel electrophoresis. The presence of sharp 28S and 18S rRNA bands, with the 28S band being approximately twice the intensity of the 18S rRNA band, and the existence of sharp and easily visible RNA bands extending up to the 10 kbp marker were the required conditions needed to consider a purified RNA sample usable in quantitative analyses.

### cDNA synthesis and two-step RT-PCR for primer validation

A two-step RT-PCR was used during the initial validation of the primers designed to amplify the different SUMO variants described in this manuscript and to clone the amplified PCR products. cDNA synthesis was performed using the M-MuLV® Reverse Transcriptase kit (New England BioLabs, Inc, Ipswich, MA) according to the manufacturer’s recommendations. The reaction mix was incubated at 42 °C for 1 h and subsequently cooled down to 4 °C. Negative control samples were produced using all the ingredients minus the M-MuLV Reverse Transcriptase; nuclease-free milli-Q water was used in place of the enzyme to keep final volumes equal. The cDNA synthesized was stored in aliquots at − 80 °C. The subsequent PCR reactions were performed using the Taq PCR kit from NEB (New England BioLabs, Inc.), using 2 μL from the RT reaction as template. The resulting PCR products were ethanol precipitated and sequenced using the Sanger method at the Genomic Analysis Core Facility, Border Biomedical Research Center, at The University of Texas at El Paso. Aliquots of the PCR products obtained were also analyzed by agarose gel electrophoresis using 1.5% agarose gels in 1 × TAE buffer (40 mM Tris, 20 mM Acetate, 1 mM EDTA, pH 8.6), and used for cloning into the pJET1.2 plasmid as described below.

### RT-qPCR

All RT-qPCR analyses were performed using the iTaqTM Universal SYBR® Green One-Step Kit from Bio-Rad (Bio-Rad Laboratories, Inc., Hercules, CA), following the manufacturer’s recommended protocol. Briefly, 100 ng of total RNA were mixed with 10 μL of Reaction Mix, 2 μL of forward primer, 2 μL of reverse primer, 0.25 μL of iScript™ Reverse Transcriptase, and nuclease-free milli-Q water up to 20 μL. All RT-qPCR were done in triplicate, so three identical reactions were set up for every sample analyzed. Negative controls were assembled using all components minus the RNA template. The RT-qPCR reactions were performed using a MyGo Pro Real-Time PCR thermocycler (Azura Genomics, Inc., Raynham, MA), and the MyGo software ran on Mac OS X platform. The thermal cycling profile used in all RT-qPCR reactions was as follows: (1) Reverse transcription step performed at 50 °C for 10 min; (2) Long denaturation at 95 °C for 3 min; (3) Two-step amplification cycles, started by denaturation at 95 °C for 10 s (ramp: 5 °C/s), followed by amplification at 60 °C for 30 s (ramp: 4 °C/s), repeated 40 times. (4) High-resolution melting curve with an initial stage of 60 °C for 1 min, a ramp of 0.05 °C/s, and a final stage of 95 °C for 1 s. To further confirm the specificity of the amplification and the validity of the data obtained, in addition to the high-resolution melting curve all RT-qPCR products obtained were analyzed on a 1.5% agarose gel, using 5 μL of the reaction.

### General molecular biology procedures

Plasmid transformations and amplifications were performed using NEB® 10-beta competent *E. coli* cells (New England BioLabs, Inc.). Maxiprep DNA purifications were performed using the ZymoPURE II Plasmid Maxiprep Kit (Zymo Research, Corp., Irvine, CA). All maxipreped DNA were quantified using a Thermo Scientific™ Invitrogen™ Nanodrop™ One Spectrophotometer (ThermoFisher Scientific, Inc.). All maxipreped DNA were diluted down to a final concentration of 1000 μg/μL and stored at − 20 °C. The quality and quantity of all maxipreped DNA was estimated by restriction analysis and agarose gel electrophoresis.

### Cloning of the products derived from the PCR amplification of the SUMO1, SUMO2, and SUMO3 transcript variants

We generated recombinant pJET1.2 plasmid constructs for each of the PCR products obtained using the primer pairs specific for each of the SUMO variants. These recombinant pJET1.2 constructs were subsequently used as templates to produce the RNA transcripts needed to generate the calibration curves to calculate copy number estimates. To generate the recombinant pJET1.2 plasmid constructs, we used the CloneJET PCR Cloning Kit (ThermoFisher Scientific, Inc.) as recommended by the manufacturer, using 1 μL of the PCR product from an RT-PCR reaction generated as indicated above. The sequence and orientation of the resulting clones was confirmed by DNA sequencing as described above.

### T7 RNA polymerase in vivo transcription

The mRNA transcripts that were used to generate calibration curves were synthesized using the pJET1.2 constructs indicated above, taking advantage of the T7-RNA Promoter located just upstream of the cloning site, and the MEGAscript™ T7 Transcription Kit (ThermoFisher Scientific, Inc.). In preparation for their use as templates, plasmids were digested using HindIII, which cuts downstream from the cloned PCR product. The digested plasmid was analyzed by gel electrophoresis to verify full digestion, and ethanol precipitated. The pellet obtained was resuspended in 20 μL of sterile TE and quantified using a Qubit Fluorometer 3.0 to ensure that exactly 1 μg of DNA would be used for in vitro transcription. The in vitro transcription reactions were performed as indicated by the manufacturer and consisted of 2 μL of each NTP, 2 μL of 10 X Reaction Buffer, 2 μL of enzyme mix, 1 μg of the HindIII-digested plasmid template, and nuclease-free milli-Q water up to 20 μL. The reaction mix was then incubated for 4 h at 37 °C. An aliquot of the resulting transcript was analyzed by gel electrophoresis to ensure that the expected product size was obtained.

### Calibration curves and CNest assessment

To obtain accurate Copy Number estimates (CNest) of each SUMO transcript variant being quantified, we generated calibration curves for each one of them. To this end, we calculated the amount of transcript in nanograms needed to have 10^10^ copies of transcript, using the transcripts synthesized using the T7 RNA Polymerase system described above. The full length of the transcript generated, and the specific nucleotide sequence of each transcript were taken into consideration to assess the molecular mass of the transcript. Once the amount of transcript needed to have 10^10^ copies was established, a dilution containing 10^9^ copies of transcript in 10 μL of buffer was made and used to generate a set of serial dilutions, each differing from its preceding dilution by a factor of 10. The lowest dilution made contained 10^3^ copies in 10 μL. The serial dilutions generated, covering the 10^3^–10^9^ copies/10 μL range, were used to set up triplicate RT-qPCR reactions using the conditions indicated above under RT-qPCR. For each transcript dilution, three independent RT-qPCR reaction were performed, the Cq values obtained were averaged, and the averages were plotted against the CNest used in each reaction. The data points obtained, corresponding to a specific Cq value for each transcript concentration, were used to generate a linear logarithmic regression that was then used to calculate CNest for each transcript variant under each experimental condition assessed. The values used for such calculations corresponded to the average Cq values from three independent experiments, each assessed in triplicate RT-qPCR reactions. The R-square, slopes, and efficiencies for all transcripts/primer-pairs are shown in Supplementary Table S3.

### Stress treatments

For stress treatments, cells were plated in 6-well plates at a concentration of 3 × 10^5^ cells per well, which provided for approximately 80% confluency by 36 h post-plating. At that time, the different stressors were applied. Three different types of stressors were used. Heat-shock was performed by placing the cells in a tissue culture incubator pre-set at 43 °C, 5% CO_2_, for 1 h. Influenza A Virus (IAV) infection was performed by washing the cells with 1 × PBS and subsequently adding media containing IAV (A/PR/8/34, H1N1) at a multiplicity of infection (MOI) of 10, and incubating the cells at 37 °C, 5% CO2, for 12 h. Cold-shock was performed by placing the cells in a tissue culture incubator pre-set at 27 °C, 5% CO_2_, for 24 h. After the indicated incubation time, the cells were washed briefly with 1 × PBS pre-equilibrated at the same temperature as the one used during the stress treatment, and the cells were subsequently lysed with 200 μL of buffer RLT and the RNA purified as indicated above. Three fully independent experiments were performed for each stress treatment for every cell type assessed. To ensure all stressors triggered the expected cellular responses, during the RT-qPCR stage we also assessed the levels of a gene transcript known to be affected by the specific stress condition being studied. Specifically, the Hsp70, Influenza M1, and Rbm3 transcripts were used as controls for heat-shock, IAV infection, and cold-shock, respectively. Additionally, to verify that the cellular stressor triggered the expected change in global cellular SUMOylation levels, a set of samples exposed to identical stress conditions were also collected for immunoblot analyses as described below.

### Nuclear vs cytosolic fractionation

Nucleocytoplasmic fractionations aimed at determining the cellular localization of transcripts were performed using the Cytoplasmic and Nuclear RNA Purification Kit from Norgen (Norgen Biotek Corporation, Thorold, ON, Canada). Briefly, cells were plated at 3 × 10^5^ cells per well in 6 well plates. At 36 h post-plating, the cells were either processed directly for cellular fractionation, or exposed to cold-shock as described above. For cellular fractionation, media was aspirated, and the cellular monolayer was washed with 2 mL of PBS. The cells were subsequently lysed by adding 200 μL of ice-cold Lysis Buffer J directly to the culture plate and gently swirling the buffer around the plate surface for five mins while keeping the plate on ice. The lysate was transferred to an RNase-free microcentrifuge tube and centrifuged for 10 min at maximum speed. The supernatant produced, containing cytoplasmic RNA, was carefully transferred to another RNAse-free tube, making sure to avoid disturbing the pellet and centrifuged once again to eliminate any potential nuclear contamination. The new cytoplasmic fraction obtained after the second centrifugation was transferred to a new tube and mixed with 200 μL of Buffer SK. The pellet left behind in both centrifugations, containing the nuclear fraction, was resuspended with 400 μL of Buffer SK. Each fraction was subsequently mixed with 200 μL of 100% ethanol, and the resulting mixes were transferred into a spin column, and centrifuged for 1 min at 3500×*g*. All subsequent steps were exactly as indicated by the manufacturer. The eluted RNA samples were stored at − 80 °C and their RNA concentrations were assessed using a Qubit Fluorometer 3.0® as indicated above. For RT-qPCR, 100 ng of the purified mRNAs were used as template, and each sample was assessed in triplicate. Three independent fractionation experiments were performed per cell line. To calculate the percentage of mRNA in each fraction, we calculated the CNest of each variant in the nuclear and cytoplasmic fraction, added them to obtain the total CNest (100%), and then calculated the percentage of each fraction by dividing the CNest of the specific fraction by the total CNest, and multiplying by 100.

### Development of plasmid constructs coding for His-S-tagged SUMO2, the His-S-tagged SUMO alphas, and the His-S-YFP-tagged SUMOs and SUMO alphas

The previously described dicistronic plasmids pcDNA5/FRT/TO/His-S-SUMO1/IRES/HA-Ubc9 and pcDNA5/FRT/TO/His-S-SUMO3/IRES/HA-Ubc9, coding for an HA-tagged Ubc9 protein (downstream cistron) and His-S-tagged SUMO1 and SUMO3, respectively (upstream cistron)^69^, were used as starting parental plasmids for all the expression plasmids used in this report. All clonings requiring the assembly of two different fragments of DNA were performed using PCR amplification of the desired fragments that needed to be “stitched” together, and Gibson assembly cloning^70^ using the NEB Gibson Assembly® Cloning Kit (New England BioLabs, Inc.). All clonings that required the PCR amplification of a single fragment followed by its re-circularization were performed using the NEB Quick Ligation™ Kit (New England BioLabs, Inc.). All PCR amplifications performed for cloning purposes were performed using the Q5® High-Fidelity DNA Polymerase from NEB (New England BioLabs, Inc.). The pcDNA5/FRT/TO/His-S-SUMO2/IRES/HA-Ubc9, coding for His-S-SUMO2, was produced by substituting SUMO2 for SUMO1 in the pcDNA5/FRT/TO/His-S-SUMO1/IRES/HA-Ubc9 construct. To this end, we used backbone-specific primers to amplify the backbone of the plasmid without amplifying SUMO1, and a PCR-amplified SUMO2 made using total RNA from HEK293A cells as template. The two PCR products were assembled together using Gibson assembly. To produce the SUMO1α and SUMO2α coding constructs, the parental plasmids indicated above, coding for the prototypical SUMOs, were used as templates and primers were designed to specifically delete the sequences eliminated during alternative splicing. The resulting PCR products were re-circularized using quick ligation. To produce the SUMO3α coding construct, primers were designed to amplify the full-length of the pcDNA5/FRT/TO/His-S-SUMO3/IRES/HA-Ubc9 plasmid and produce a linear product with ends located around the region where the additional sequence is introduced by alternative splicing of the transcript. The additional sequence, corresponding to the intronic extension of exon 2, was produced by using two long oligonucleotides covering the desired additional sequence and providing for two overlaps, one with the ends of the PCR-amplified linearized parental construct, and one with each other. The two primers were designed to run in anti-parallel directions, and the overlap with each other was limited to 30 bases at their 3’ ends. The hybridized long oligonucleotides were used as templates for a PCR reaction that included additional forward and reverse primers, which targeted the ends of the templates in anti-parallel direction. Such PCR reaction generated a product ready for Gibson assembly with the PCR-linearized parental plasmid. The His-S-YFP-tagged constructs were developed by PCR-amplifying the entire sequence of the parental clones using primers targeting the sequence located downstream of the His-S-tag sequence. As those sequences were shared by all the parental clones, the same set of primers were used in all of the amplifications. The coding sequence for YFP was amplified using the pEYFP plasmid (Addgene, Watertown, MA) as template. The PCR products corresponding to the linearized parental clones and the YFP coding sequence were stitched together in independent reactions (one per parental plasmid) using the Gibson assembly method. All the recombinant plasmids generated were amplified in NEB® 10-beta *E. coli* cells and their sequence confirmed by DNA sequencing as above. Detailed information related to the cloning methods used is available upon request.

All recombinant DNA protocols, including the use of IAV, were approved by the Institutional Biosafety Committee (IBC) at The University of Texas at El Paso (UTEP).

### Immunoblot analyses

For immunoblot analyses of cells exposed to different stressors, cells were plated and treated as described above under “stress treatments” and collected in boiling 4 × Laemmli Sample Buffer as described below. For immunoblot analyses of cells expressing the His-S-tagged prototypical SUMO or SUMO alpha proteins, HEK293A cells were plated in 12 well plates at 1 × 10^5^ cells per well in 1.5 mL of 1 × Complete Medium. The cells were grown at 37 °C, 5% CO_2_ for 24 h and transfected with the indicated plasmid. Transfection mixes were prepared by diluting 4 μg of plasmid DNA (at a concentration of 1 μg/μL) in 184 μL of Opti-MEM™ I (Gibco™, ThermoFisher Scientific, Inc.), and adding 12 μL of Trans-IT® LT1 transfection reagent (Mirus Bio, LLC, Madison, WI). The transfection mix was allowed to sit undisturbed for 20 min at room temperature and subsequently added directly to the cells, without changing the medium. Upon transfections the cells were grown for 26 h at 37 °C, 5% CO_2._ The transfected cells were collected by discarding the medium using vacuum suction, washing gently with 1 × PBS (pre-warmed to 37 °C) for about 1 min, discarding the 1 × PBS, and adding 500 μL of boiling 4 × Laemmli Sample Buffer directly to the cells. The resulting cell extract was transferred to a 1.5 mL microcentrifuge tube and passed through a 29½ gauge needle, using tuberculin syringes to shear all genomic DNA and prevent artifacts during the SDS-PAGE. In preparation for SDS-PAGE, all samples were treated with 50 μL of β-mercaptoethanol and boiled for 5 min. For SDS-PAGE, 30 μL per sample were run on a 14 cm × 12 cm × 0.15 cm discontinuous 10% SDS-PAGE gel, using a 15 well-comb, at 50 Volts overnight, on a Hoefer™ SE 600 Series Vertical Electrophoresis System (Fisher Scientific, ThermoFisher Scientific, Inc.). After electrophoresis, the gel was equilibrated in 1 × Transfer Buffer (20% Methanol, 25 mM Tris, 192 mM Glycine, pH 8.3) for 10 min at room temperature and proteins transferred to a PVDF membrane using the wet-transfer method at 1.6 mA for 2 h 50 min using an Owl™ VEP-3 Large Tank Electroblotting System (ThermoFisher Scientific, Inc.). Upon transfer, the PVDF membranes were allowed to dry overnight, re-wetted in absolute methanol, washed 3 times in milli-Q water, and washed two additional times with 1 × PBS. The PVDF membranes were blocked in 1 × Blocking Solution (1 × PBS + 3% fat-free milk + 0.1% Tween 20), for 1 h at room temperature. Incubation with primary antibodies was performed over-night at 4 °C. Subsequently, the membranes were washed with 1 × TPBS (1 × PBS + 0.1% Tween 20) for 3 min, 3 times, and incubated with the secondary antibodies in 1 × Blocking Solution for 1 h at room temperature. In preparation for development, membranes were washed 3 times with 1 × TPBS and 1 time with 1 × PBS. To develop the immunoblots, the membranes were soaked on SuperSignal™ West Pico PLUS Chemiluminescent Substrate solution (Fisher Scientific, ThermoFisher Scientific, Inc.) and images were captured using an iBright™ FL1500 Imaging System (ThermoFisher Scientific, Inc.).

The primary and secondary antibodies used were as follows:

SUMO1: Given the limitations inherent to the use of single antibodies in the detection of SUMO1 conjugates^71^, a cocktail of three different antibodies was used, rabbit polyclonal anti-SUMO1 Y299 from Abcam (Abcam, Cambridge, UK), 1:5,000 dilution; rabbit monoclonal anti-SUMO 1 C9H1 from Cell Signaling (Cell Signaling Technology, Inc., Danvers, MA), 1:5,000 dilution; and, a previously reported rabbit polyclonal serum against SUMO-1 developed in house (#12783)^45^, 1:3,000 dilution.

SUMO2: Rabbit polyclonal anti-SUMO2 (Sentrin 2) from Zymed (51-9100)(Zymed Technologies, ThermoFisher Scientific, Inc.), 1:3,000 dilution.

S-tag: Mouse monoclonal anti S-Tag, clone GT247, from Sigma (Sigma-Aldrich, MilliporeSigma, St. Louis, MO), 1:5,000 dilution.

GAPDH: Rabbit monoclonal anti-GAPDH (14C10), from Cell Signaling (Cell Signaling Technology, Inc.), 1:5,000 dilution.

Secondary anti-rabbit: Mouse anti-rabbit IgG-HRP conjugated (sc-2357), from Santa Cruz Biotech (Santa Cruz Biotechnology, Inc., Dallas, TX), 1:5,000 dilution.

Secondary anti-mouse: Goat anti-mouse IgG-HRP conjugated (AP181P), from Sigma (MilliporeSigma), 1:5,000 dilution.

### Confocal microscopy

For confocal microscopy, HEK293A cells were plated at 1 × 10^4^ cells well, using 100 μL of 1 × Complete Medium. The cells were grown at 37 °C, 5% CO_2_ for 24 h and transfected with the indicated plasmid. Transfection mixes were prepared by diluting 5 μg of plasmid DNA (at a concentration of 1 μg/μL) in 380 μL of Opti-MEM™ I (Gibco™, ThermoFisher Scientific, Inc.), and adding 15 μL of Trans-IT® LT1 transfection reagent (Mirus Bio). The transfection mix was allowed to sit undisturbed for 20 min at room temperature and subsequently 40 μL of the mix were added directly to each well, without changing the medium. Upon transfections, the cells were grown for 24 h at 37 °C, 5% CO_2_.

In preparation for confocal microscopy, the cells were fixed by removing the culture media and immediately adding 100 μL of 1 × PBS + 4% Formaldehyde and incubating for 10 min. The cells were subsequently permeabilized with 200 μL of 1 × TPBS and stained for 1 h at room temperature, in the dark, with 25 μL of 1 × Staining Solution. The 1 × Staining Solution was made by mixing 10 μL of 66 μM Alexa-Fluor 568-Phalloidin (ThermoFisher Scientific, Inc.), 10 μL of 1 μg/mL DAPI (4',6-Diamidino-2-Phenylindole, Dihydrochloride) (ThermoFisher Scientific, Inc.), 80 μL of 1 × PBS + 5% BSA, and 300 μL of 1 × PBS. Subsequently, the cells were washed once with 200 μL of 1 × TPBS, and once with 200 μL of 1 × PBS. Confocal microscopy images were obtained with a Zeiss LSM 700 confocal microscope system (Zeiss, New York, NY) using a Plan-Apochromat 20x/0.8 air objective. For every set of images captured, three different lasers were used, a 488 nm laser for YFP imaging (green, YFP-tagged SUMO proteins), a 496 nm laser for Phalloidin imaging (red, actin filaments), and a 405 nm laser for DAPI imaging (blue, DNA). The power of all lasers used was set at 5% with an airy unit pinhole setting of 1. The gain settings were 577 for DAPI, 582 for Phalloidin, and 377 for GFP; these settings were used consistently for all images captured. Image processing and analysis were performed using the ZEN 2009 software (Zeiss, New York, NY).

### In-silico identification of SUMO alpha patterns in Ribo-seq datasets

To seek for SUMO alpha-specific transcript sequences in existent Ribo-seq data repositories, five datasets, selected at random among those availables, were downloaded as gene expression profiles (fastq sequences) from the Sequence Read Archive (SRA) database (https://www.ncbi.nlm.nih.gov/sra). The accession numbers for those datasets are SRP314256, SRP308047, SRP122522, SRP362491, and SRP286677. The fastq files associated with these datasets were retrieved in batches using the SRA toolkit, prefetch, fastq-dump and python. The SRA toolkit commands were incorporated into python code and the files were retrieved. The fastq files were searched for the presence of specific SUMO alpha transcript sequences using the SeqKit tool^72^. The criteria for positivity required the entire sequence of the matched segment to be identical to that of the query sequence used. If the sequence match was longer than the length of the query, the additional nucleotides had to match the extended sequence of the query (that is, including additional 5’ and 3’ sequences that surround the one used as query).

### Tertiary structure prediction analyses

Homology-based structural predictions were performed using the web-based RaptorX prediction software hosted at the University of Chicago (http://raptorx.uchicago.edu/StructPredV2/predict/)^73^. Ad initio modelings were performed using Alpha Fold v2.0 system, downloaded from its open source repository at https://www.deepmind.com/open-source/alphafold^74^. The tertiary structures generated for each SUMO alpha protein using the methods above were saved as “.pdb” files (protein data bank file) and viewed using UCSF Chimera, downloaded from its University of California at San Francisco repository, at https://www.cgl.ucsf.edu/chimera/download.html.

### Statistical analyses

Copy Number estimates (CNest) were calculated using the calibration curves generated as described above by entering the average Cq values obtained in triplicate experiments, each measured in triplicate RT-qPCR reactions. For stress treatments, the average differences in CNest obtained between positive and negative treatments were compared using an unpaired Student’s T-Test. A Bonferroni correction was conducted to correct for the number of multiple comparisons within each treatment (significance: p < 0.008). Nuclear and Cytosolic cellular fractions were compared using the log2 scale of the 2-∆CT method. All analyses were conducted using Stata v.17 and GraphPad Prism V.6.0.

All methods described above, as well as all the research described in this report, were performed according to the rules and regulations for biological and laboratory safety and recombinant DNA work set by the Institutional Biosafety Committee (IBC), the Institutional Review Board (IRB) Committee, and the Environmental Health and Safety (EH&S) Department, all at The University of Texas at El Paso (UTEP).

## Supplementary Information

Supplementary Information.

## Data Availability

The Excel sheets containing all the data reported in this manuscript, as well as all the expression plasmids herein reported, are available upon request. Please direct all requests to the Corresponding Author, Dr. Rosas-Acosta, at grosas3@utep.edu.
