# Supplementary material for: Alternative splicing of the SUMO1/2/3 transcripts affects cellular SUMOylation and produces functionally distinct SUMO protein isoforms
Source: Sci Rep. 2023 Feb 9;13:2309. doi: 10.1038/s41598-023-29357-7 (PMC9911741; doi:10.1038/s41598-023-29357-7)
Supplement: Supplementary file 1 — Supplementary Information. [file 41598_2023_29357_MOESM1_ESM.pdf]

Alternative splicing of the SUMO1/2/3 transcripts affects cellular SUMOylation and produces functionally distinct SUMO protein isoforms

AUTHORS:

Myriah L. Acuña[1,+], Andrea García-Morin[1,+], Rebeca Orozco-Sepúlveda[1], Carlos Ontiveros[1], Alejandra Flores[1], Arely V. Diaz[1], Isabel Gutiérrez-Zubiate[1], Abhijeet R. Patil[2], Luis A. Alvarado[3], Sourav Roy[1,4], William K. Russell[5], Germán Rosas-Acosta[1,4,*]

SUPPLEMENTARY FIGURES AND TABLES

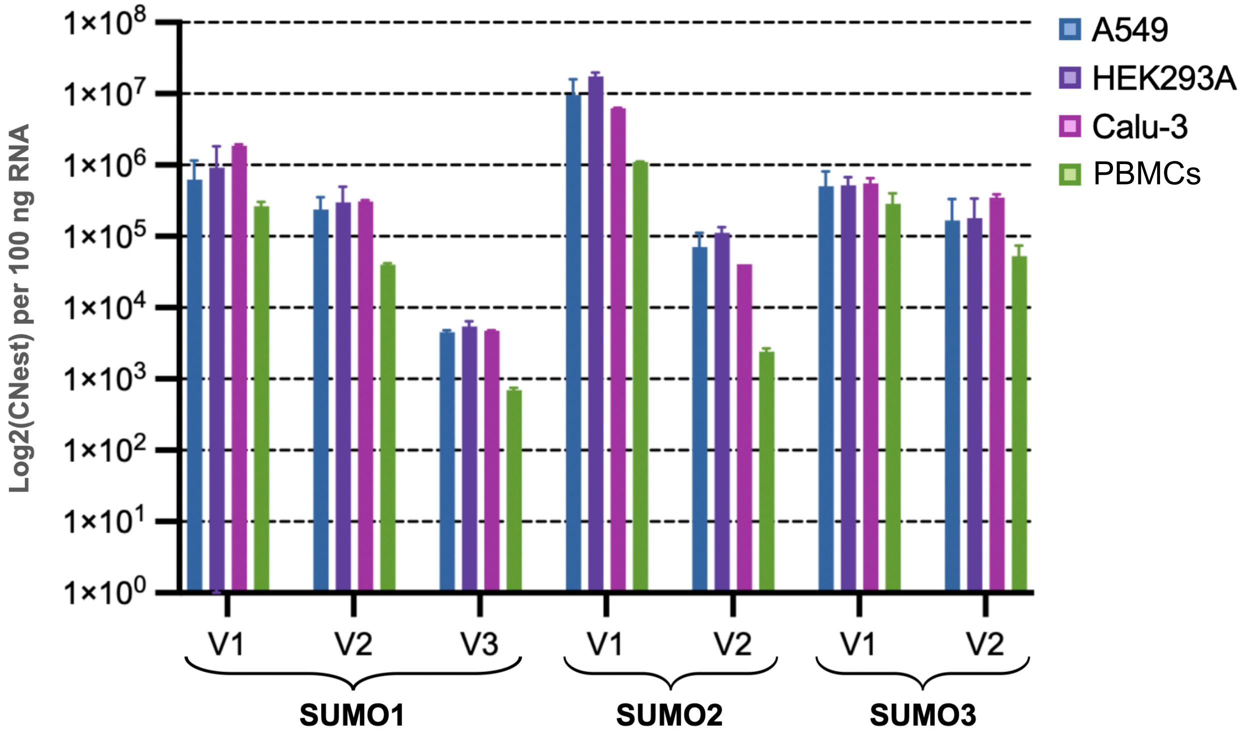

Supplementary Figure S1. Copy Number estimates (CNest) per 100 ng of RNA of all SUMO transcripts analyzed in this study in A549, HEK293A, and Calu-3 cells, as well as in PBMCs from healthy volunteers, under normalcy. All data represents the average values obtained from triplicate measurements in three independent experiments. Error bars indicate standard deviation. No statistical comparisons were made among different groups as the goal pursued was to simply establish what the normal levels were for all transcripts presented under normal conditions. This data corresponds to the same data represented in Figure 3.

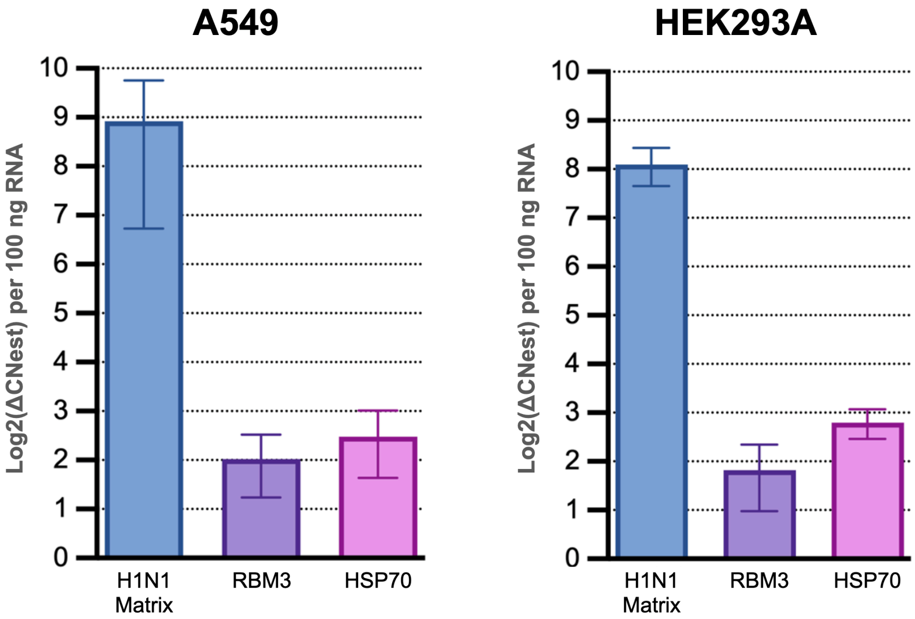

Supplementary Figure S2. Changes in the relative abundance of the mRNA transcripts used as controls for the different stress conditions. A549 cells and HEK293A cells were exposed to three different types of stress, namely IAV (Influenza A virus infection using viral strain A/PR/8/34 H1N1 at MOI=10 for 12 hours), cold-shock (27°C for 24 hours), and heat-shock (43°C for 1 hour), as described under Materials and Methods. Untreated cell cultures plated at equal cell densities and maintained for equal time frames were used as controls. After treatment, total RNA was purified and the CNest for the specific transcript shown was calculated. Changes in relative abundance of each transcript were calculated by subtracting the CNest in the control (non-stressed sample) from the CNest value obtained upon stress. All data are shown in Log2 scale and represent the average values obtained from triplicate measurements in three independent experiments. The H1N1 Matrix gene was used as control for IAV infection, RBM3 was used as control for cold-shock, and Hsp70 was used as control for heat-shock.

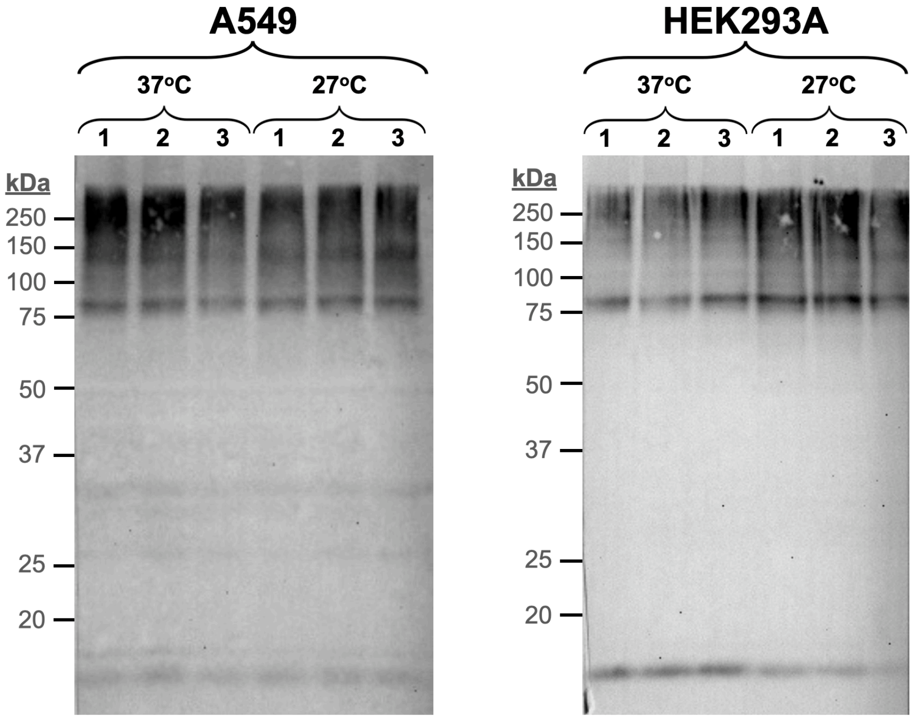

Supplementary Figure S3. Changes in global SUMOylation levels triggered by cold-shock in A549 and HEK293A cells. To confirm the differences in the global SUMOylation levels observed upon cold-shock in A549 cells as compared with HEK293A cells, we performed three independent cold-shock experiments. In each, cells were grown at either 37°C or 27°C for 24 hours and collected and processed for immunoblotting analyses using anti-SUMO1 antibodies as described under Materials and Methods. 37°C: Samples grown at 37°C (normal conditions); 27°C: Samples grown at 27°C. In each case, lanes 1-3 represent samples from a different independent experiment.

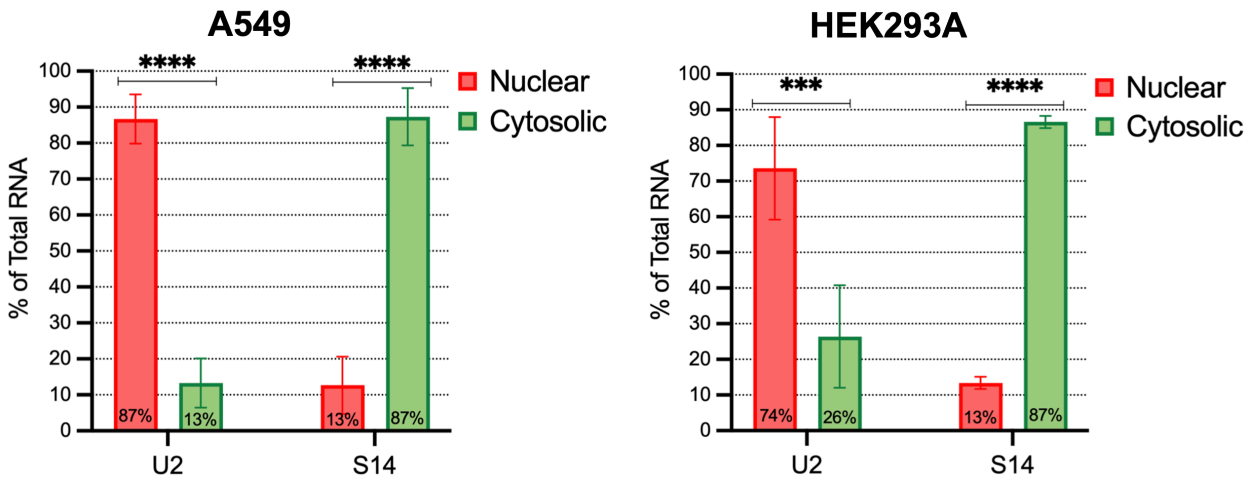

Supplementary Figure S4. Nucleocytoplasmic distribution of U2 snRNP RNA and the mRNA derived from the gene coding for the ribosomal protein S14, which are the control RNAs used for cellular fractionation experiments. Cells grown at 37°C for 24 hours were collected, lysed, & separated into nuclear and cytoplasmic fractions. Total RNA was purified from each fraction and used to estimate nuclear and cytoplasmic CNests for each RNA. The sum of the nuclear and cytoplasmic fraction equals 100% of the total transcript present in the cell for that RNA. The data presented correspond to the average values from triplicate measurements obtained in three independent experiments. Statistical significance was established using an unpaired Student's T-Test, applying a Bonferroni correction to account for the number of multiple comparisons within each treatment. ***$p<0.002$; ****$p<0.001$.

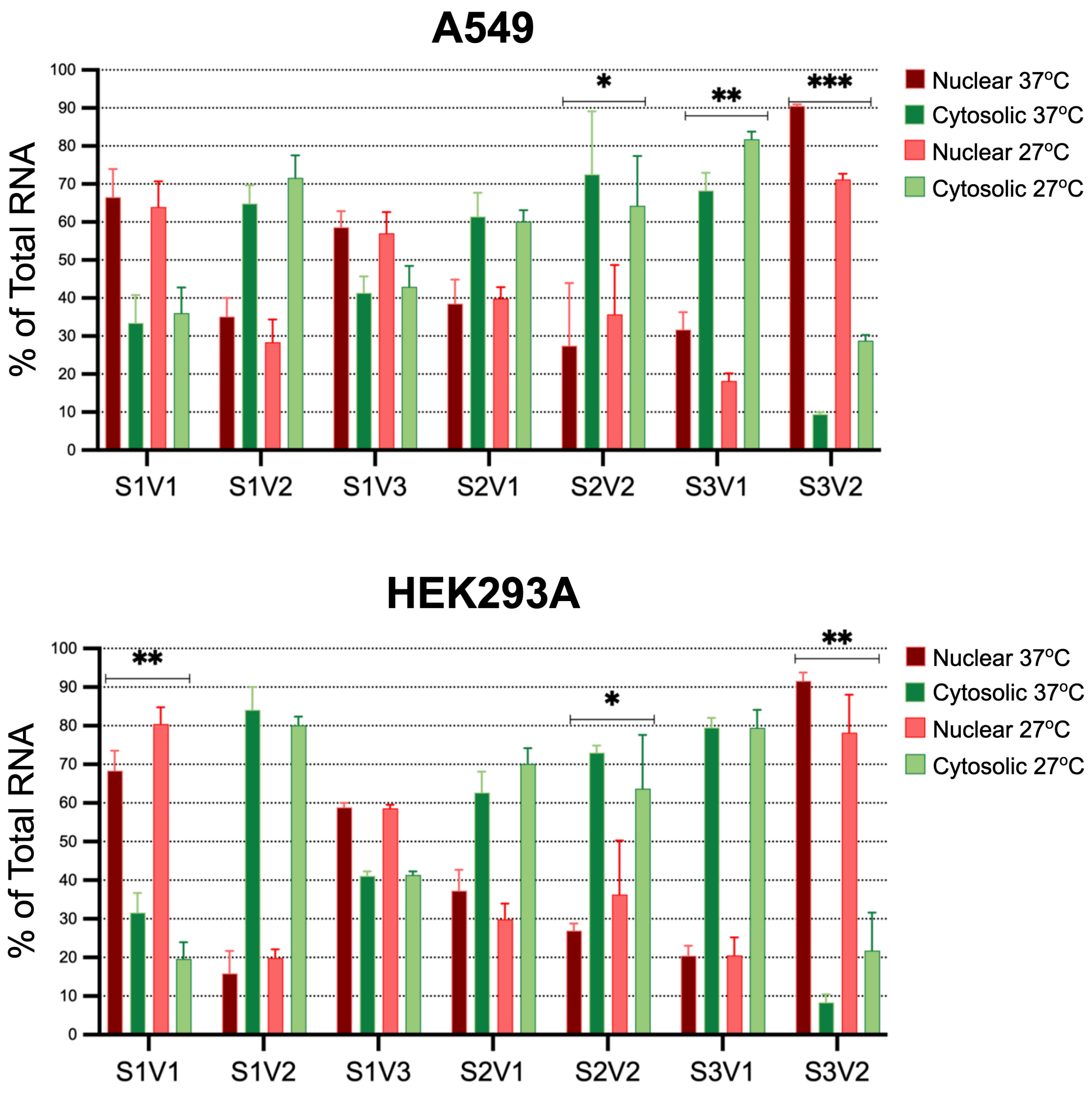

Supplementary Figure S5. Nucleocytoplasmic distribution of each variant under normalcy (37°C) and cold-shock (27°C) in A549 and HEK293A cells. Cells maintained for 24 h at either 37°C or 27°C (cold-shock) were collected, lysed, & separated into nuclear and cytoplasmic fractions. Total RNA was purified from each fraction and used to estimate nuclear and cytoplasmic CNests for each variant. The data presented correspond to the average values from triplicate measurements obtained in three independent experiments. In each case, the sum of the nuclear and cytoplasmic fraction equals 100% of the total transcript present in the cell for that variant. Error bars indicate standard deviation. Statistical significance was established using an unpaired Student's T-Test, applying a Bonferroni correction to account for the number of multiple comparisons within each treatment. *$p<0.008$; **$p<0.004$. ***$p<0.002$. This data corresponds to the same data presented in Figure 5A.

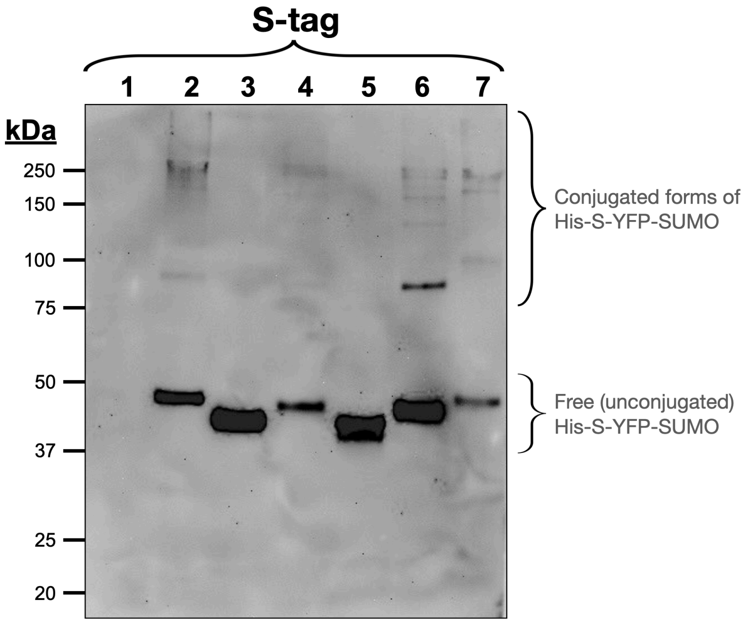

Protein	Expected MW (kDa)
**His-S-YFP-SUMO1**	43.85
**His-S-YFP-SUMO1α**	41.09
**His-S-YFP-SUMO2**	43.32
**His-S-YFP-SUMO2α**	40.83
**His-S-YFP-SUMO3**	43.13
**His-S-YFP-SUMO3α**	47.26

Supplementary Figure S6. Immunoblotting profile of His-S-YFP-SUMO fusion proteins used for immunofluorescence analyses. HEK293A cells were transfected with expression constructs coding for the indicated His-S-YFP-tagged SUMO protein. At 24 h post-transfection, the transfected cells were collected in boiling 4x Sample Buffer and processed for SDS-PAGE and immunoblotting analysis using antibodies against the S-tag, which is located between the His-tag (itself located near the N-terminus of the protein) and the YFP tag. The localization of free (unconjugated) and conjugated SUMO forms are indicated. 1, Cells transfected with an empty plasmid; 2, cells over-expressing His-S-YFP-SUMO1; 3, cells over-expressing His-S-YFP-SUMO1α; 4, cells over-expressing His-S-YFP-SUMO2; 5, cells over-expressing His-S-YFP-SUMO2α; 6, cells over-expressing His-S-YFP-SUMO3; 7, cells over-expressing His-S-YFP-SUMO3α. Notice the absence of truncated forms for all of the fusion proteins expressed, which indicates that the localization data provided in Figure 7 corresponds to the actual cellular distribution dictated by the corresponding SUMO protein. The expected molecular weights of the different fusion proteins are shown in the table provided. Notice also the lack of conjugated forms for His-S-YFP-SUMO1α and His-S-YFP-SUMO2α, which supports the conclusion that the alpha isoforms of SUMO1 and SUMO2 are not conjugatable (see Fig. 8D).

Supplementary Table S1. Primers used in this study.

GENE	mRNA transcript (variant)	Size of PCR product	Primer #	Primer Name	Primer Orientation FW	Primer Orientation RV	Sequence	Location	Primer Use (mRNA targeted and/or experimental goal pursued)
SUMO1	SUMO1V1	345 bp	1	S1V1V2.FW	X		5'-ACTCAAAGTCATTGGACAGGATAGCAGTGAG-3'	Exon1/Exon2 junction. Present in S1V1 & S1V2, but not in S1V3.	SUMO1V1 & SUMO1V2
			2	S1V1V3.RV		X	5'-AGAGATGGGGTGCCAGTTTTCAATTCC-3'	Cryptic intron within Exon 5, spliced out in S1V2. Present in S1V1 and S1V3, but not in S1V2.	SUMO1V1 & SUMO1V3
	SUMO1V2	258 bp		S1V1V2.FW	X		See primer #1 above	See primer #1 above	See primer #1 above
			3	S1V2.RV		X	5'-CCAGACCCTCAAATTTTAAAACTAAACTGTTGAATGACCC-3'	Exon5/Exon5' Junction produced by splicing of cryptic intron in Exon 5. Exclusive of S1V2.	SUMO1V2
	SUMO1V3	344 bp	4	S1V3.FW	X		5'GTCATCATGTCGACCAGGATAGCAGTG-3'	Exon1/Exon3 junction. Exclusive of S1V3.	SUMO1V3
				S1V1V3.RV		X	See primer #2 above	See primer #2 above	See primer #2 above
SUMO2	SUMO2V1	241 bp	5	S2V1V2.FW	X		5'-ACGAAAAGCCCAAGGAAGGAGTCAAG-3'	Exon1/Exon2 junction. Present in S2V1 & S2V2.	SUMO2V1 & SUMO2V2
			6	S2V1.RV		X	5'-TGTATCTTCATCCTCCATTTCCAACTGTGC-3'	Exon3/Exon4 junction. Exclusive of S2V1.	SUMO2V1
	SUMO2V2	169 bp		S2V1V2.FW	X		See primer #5 above	See primer #5 above	See primer #5 above
			7	S2V2.RV		X	5'-TGTATCTTCATCCTCCATTTCCAACTGTCG-3'	Exon2/Exon4 junction. Exclusive of S2V2.	SUMO2V2
SUMO3	SUMO3V1	179 bp	8	S3V1.FW	X		5'-TGCGAGAGGCAGGGCTTGTCAATGAGGC-3'	Exon2/Exon3 junction. Exclusive of S3V1.	SUMO3V1
			9	S3V1V2.RV		X	5'-GGCCCTCTAGAAACTGTGCCCTGCCAG-3'	Exon4, Present in S3V1 & S3V2.	SUMO3V1 & SUMO3V2
	SUMO3V2	239 bp	10	S3V2.FW	X		5'-CTGGTCCTGTGCGTTCCAGGCATCCC-3'	Intronic sequence retained as part of Exon 2 in S3V2. Exclusive of S3V2.	SUMO3V2
				S3V1V2.RV		X	See primer #9 above	See primer #9 above	See primer #9 above
Control genes	M1 - IAV	233 bp	11	Matrix.FWD	X		5'-CCGCAAACGAGTTGCAGACC-3'	M gene segment - IAV genome	IAV Infection Control
			12	Matrix.RV		X	5'-CTTCTGACCTAATTGTTCCCGCC-3'	M gene segment - IAV genome	IAV Infection Control
	RBM3	221 bp	13	RBM3.FW	X		5'-TGGTCGCAGCTACTCTAGAGGTGG-3'	RBM3 gene transcript	Cold-Shock Control
			14	RBM3.RV		X	5'-GGATCAGAAATTATTCCTTGTGTATCTATATTATGTGC-3	RBM3 gene transcript	Cold-Shock Control
	HSP70	151 bp	15	HSP70.FW	X		5' GCGTGATGACTGCCCTGAT-3'	Hsp70 gene transcript	Heat-Shock Control
			16	HSP70.RV		X	5'-CGCCCCAACAGATTGTTGT-3'	Hsp70 gene transcript	Heat-Shock Control
	U2	169 bp	17	U2.FW	X		5'-CATCGCTTCTCGGCCTTTTG-3'	snRNP-U2 RNA	Nuclear RNA Control
			18	U2.RV		X	5'-TGGAGGTACTGCAATACCAGGC-3'	snRNP-U2 RNA	Nuclear RNA Control
	S14	166 bp	19	S14.FW	X		5'-GGCAGACCGAGATGAATCCTC-3'	S14 Ribosomal gene transcript	Cytosolic mRNA Control
			20	S14.RV		X	5'-CAGGTCCAGGGGTCTTGGTCC-3'	S14 Ribosomal gene transcript	Cytosolic mRNA Control

Supplementary Table S2. Predicted tryptic cleavage products for the prototypical SUMO proteins and their SUMOα isoforms

	Prototypical SUMO (isoform 1)	SUMOα (isoform 2)	Sequence of "alpha-specific" tryptic fragments
**SUMO1**	2579.1453	2579.1453	
	1750.7799	1423.6158	MSDQDSSEIHFK
	1359.6903	1001.5197	
	1001.5197	896.4624	
	896.4624	895.4632	
	895.4632	785.3247	
	785.3247	730.3916	
	738.3668		
	730.3916		
**SUMO2**	3830.6861	2445.0762	QLEMEDEDTIDVFQQQTGGVY (C-end sequence)
	1234.6426	1234.6426	
	1197.5858	1197.5858	
	818.4076	818.4076	
	691.3556	682.3882	
	682.3882	641.2712	
	641.2712		
**SUMO3**	4680.0841	4680.0841	
	1234.6426	2280.2718	HLAPPQSLPVCALVLCVPGIPR
	1083.5429	1633.7825	GWTQMQLPEGLSMR
	848.4182	1234.6426	
	691.3556	1083.5429	
	682.3882	848.4182	
	641.2712	682.3882	
		641.2712	

Peptides specific of the prototypical SUMOs (isoform 1) are shown in aqua background. Peptides specific of the SUMOα (isoform 2) are shown in yellow background. The predictions were performed using the PeptideMass tool at the ExPASy Bioinformatics Resource Portal (http://web.expasy.org/cgi-bin/peptide_mass/peptide-mass.pl).

Supplementary Table S3. Efficiencies of the primer pairs used for RT-qPCR analyses.

Transcript Variant	Forward Primer	Reverse Primer	R Square	Slope	Efficiency
**SUMO1V1**	S1V1V2.FW	S1V1V3.RV	0.99	-3.0548	112.4974
**SUMO1V2**	S1V1V2.FW	S1V2.RV	0.9873	-3.0143	114.6604
**SUMO1V3**	S1V3.FW	S1V1V3.RV	0.9947	-3.3573	98.5447
**SUMO2V1**	S2V1V2.FW	S2V1.RV	0.994	-3.2983	100.9955
**SUMO2V2**	S2V1V2.FW	S2V2.RV	0.9949	-3.3675	98.1327
**SUMO3V1**	S3V1.FW	S3V1V2.RV	0.9968	-3.4914	93.3828
**SUMO3V2**	S3V2.F2	S3V1V2.RV	0..9859	-3.2029	105.2187

The R-Square and Slope values shown correspond to the numbers obtained in the calibration curves performed for every PCR product using the indicated primer pairs.

ADDITIONAL SUPPLEMENTARY FIGURES - ORIGINAL UNCROPPED IMAGES

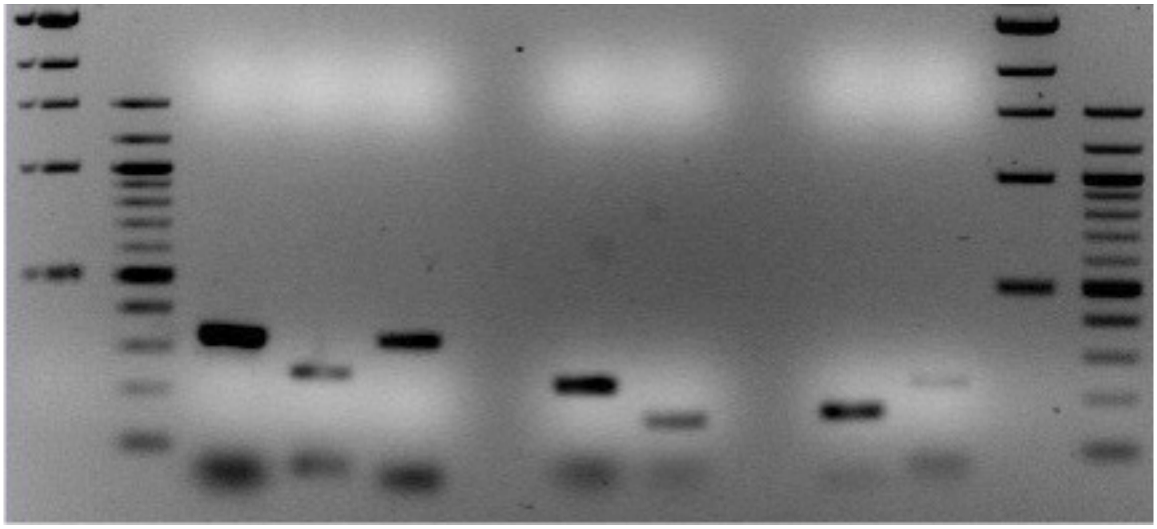

FIGURE 2 (b)

RT-qPCR products obtained for each mRNA variant form using the primer pairs represented in A. All products obtained were sequenced to confirm their identity and the specificity of the reaction.

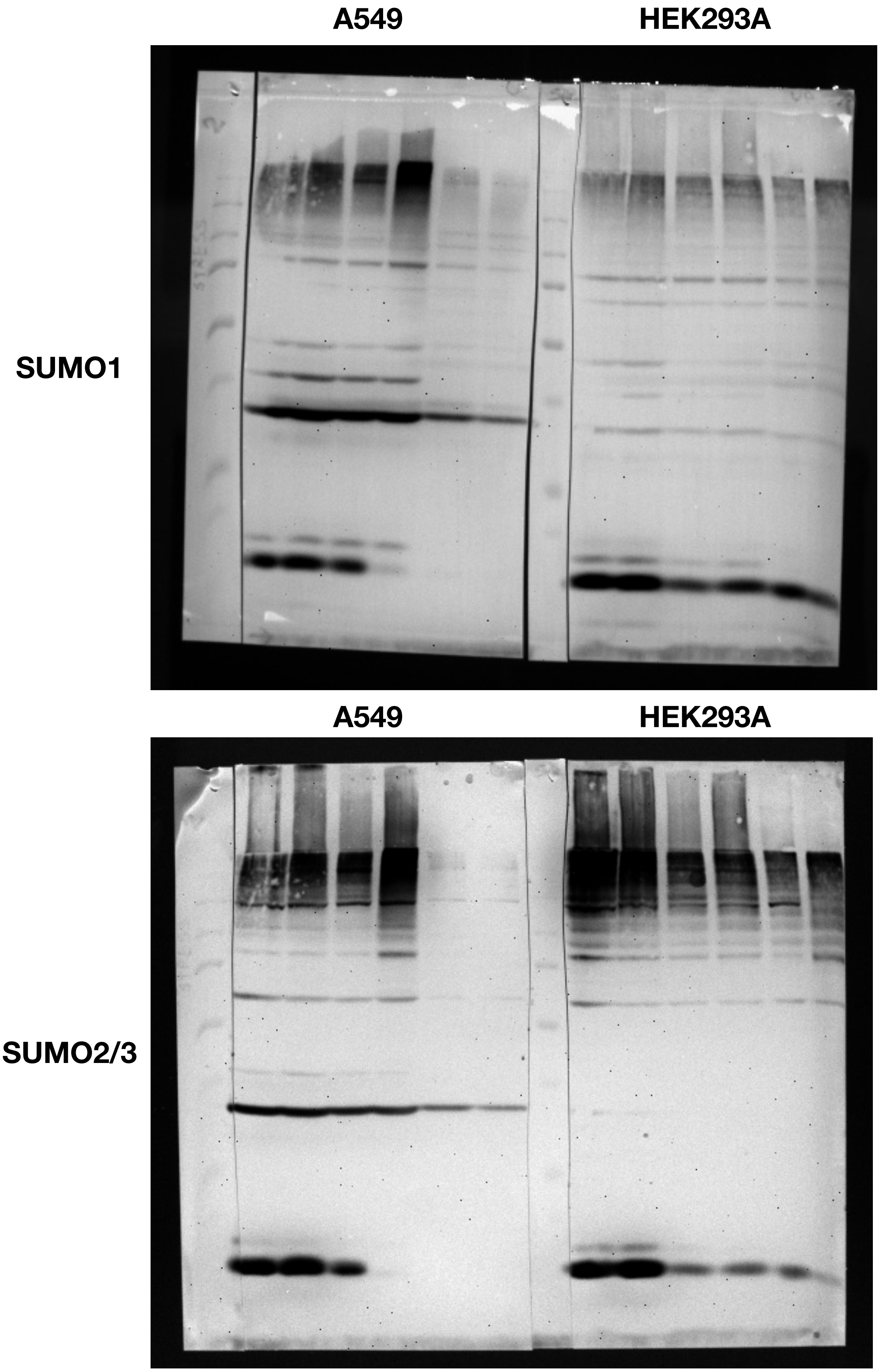

FIGURE 4 (d)

A549 **HEK293A**

GAPDH

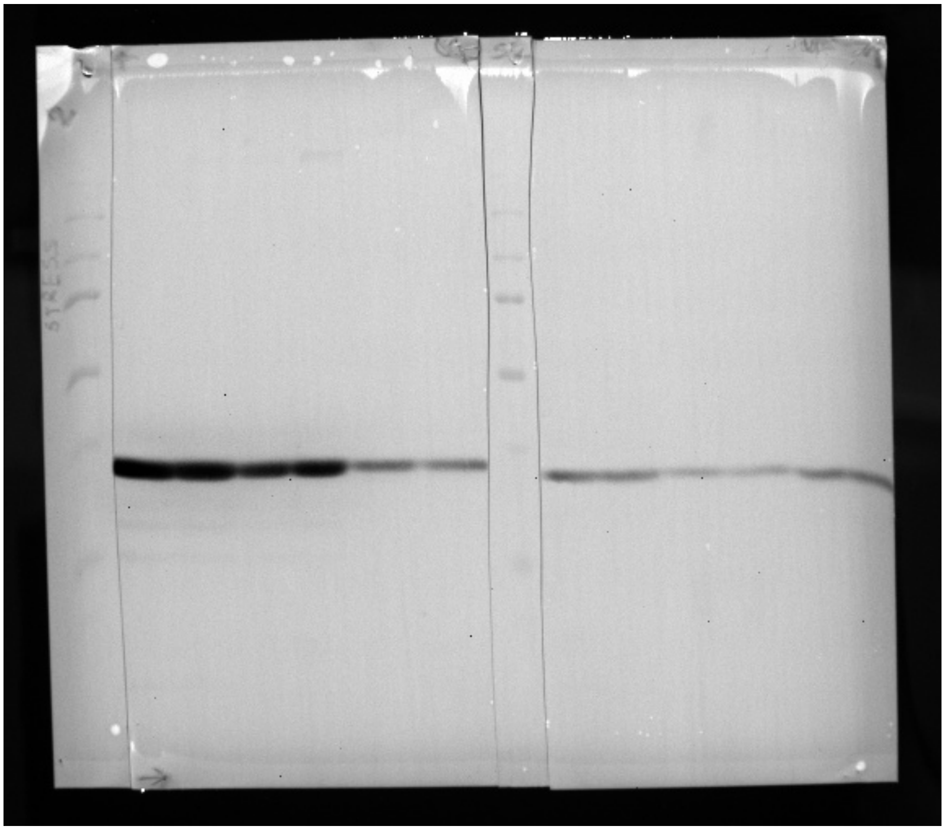

FIGURE 4 (d) (Continuation)

Immunoblot data showing changes in global SUMOylation levels triggered by Influenza A Virus infection, heat- shock, and cold-shock. The membranes were immunoblotted for GAPDH, washed, immunoblotted for SUMO1, stripped using a heat denaturation method, and subsequently blotted for SUMO2/3. 1, Mock infected cells; 2, IAV infected cells; 3, heat-shock control; 4, heat-shock sample; 5, cold-shock control; 6, cold- shock sample. *: GAPDH.

S-tag **GAPDH**

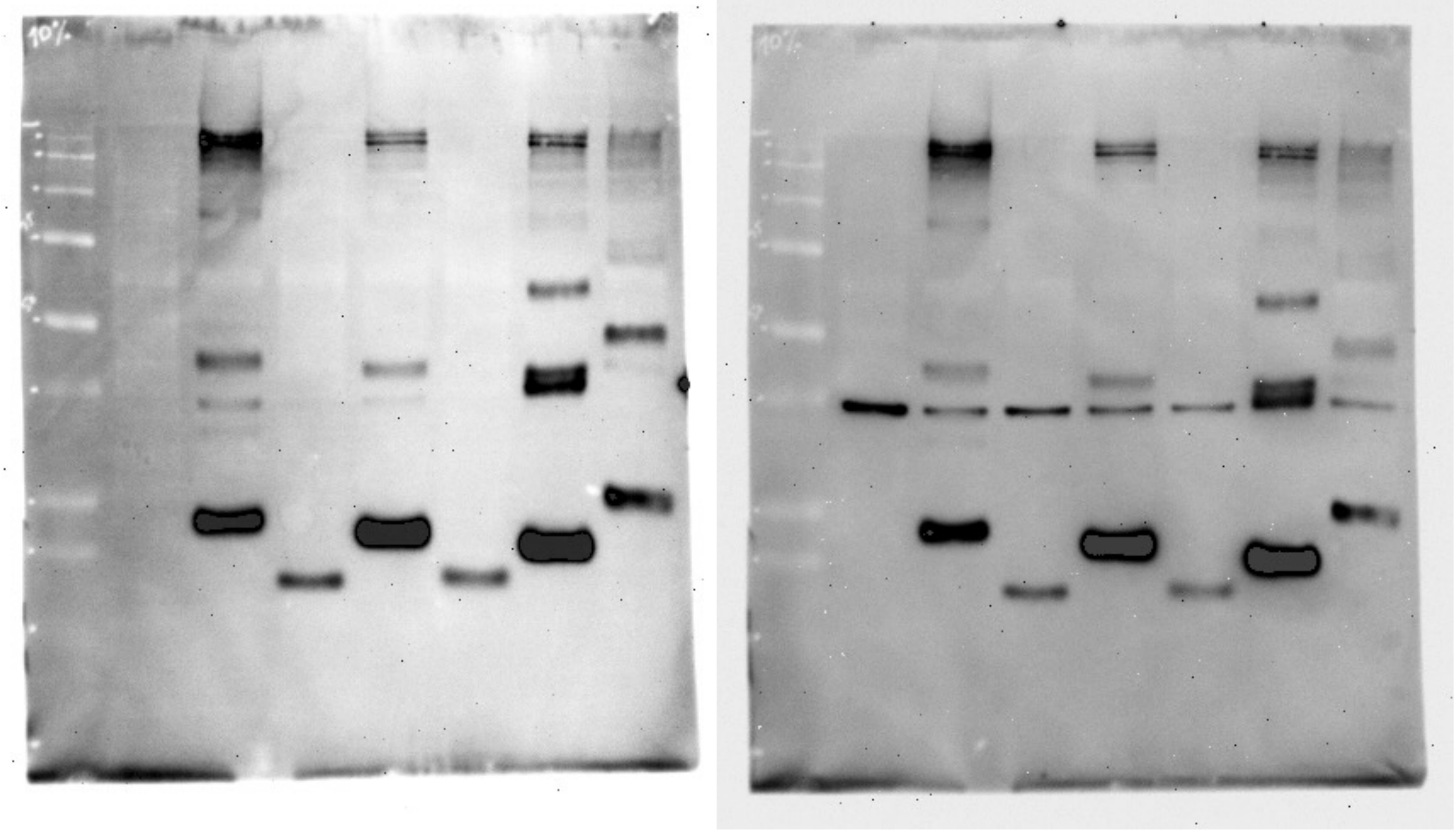

FIGURE 8 (d)

Immunoblot analyses showing the patterns of conjugation associated to each SUMO isoform. HEK293A cells were transfected with expression constructs coding for the indicated His-S-tagged SUMO protein. At 24 h post-transfection, the cells were lysed and the resulting extracts analyzed by SDS-PAGE and immunoblotting. Upper panel: immunoblot performed with antibodies directed against the S-tag (located near the N-terminal end of the proteins). Lower panel: subsequent immunoblot using anti-GAPDH antibodies as loading control for the different samples. The localization of free- and conjugated-SUMO forms is indicated. High molecular weight signals are easily visible for SUMO3α, indicating its ability to become conjugated to other proteins. In contrast, no high molecular weight forms were observed for SUMO1α and SUMO2α. 1, Mock transfected cells; 2&3, cells over-expressing SUMO1 and SUMO1α, respectively; 4&5, cells over-expressing SUMO2 and SUMO2α, respectively; 6&7, cells over- expressing SUMO3 and SUMO3α, respectively. All over-expressed SUMO proteins contained tandem His- and S-tags at their N- terminus.

A549

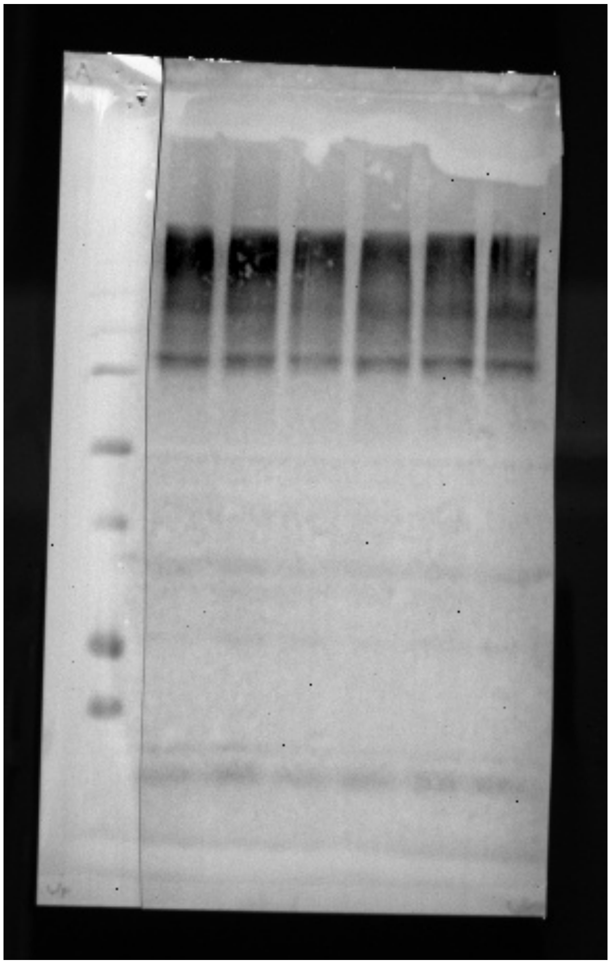

HEK293A

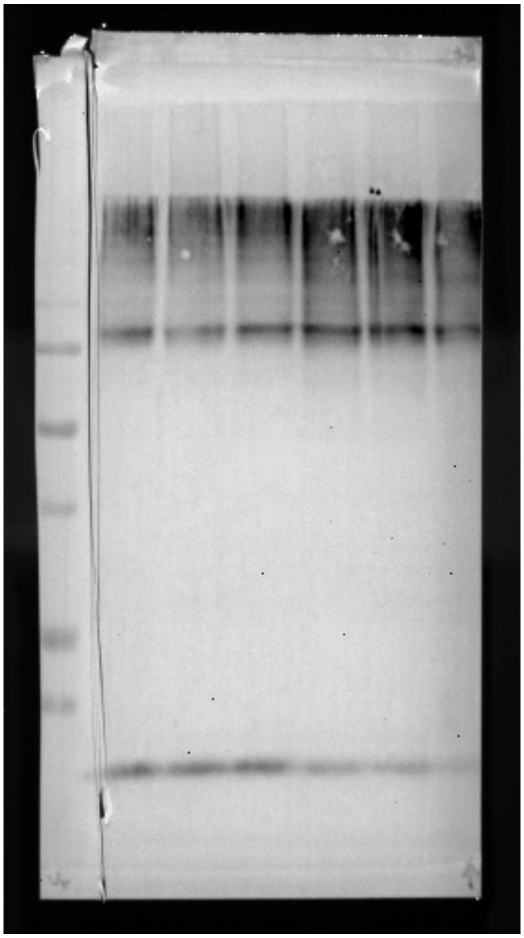

Supplementary Figure S3

To confirm the differences in the global SUMOylation levels observed upon cold-shock in A549 cells as compared with HEK293A cells, we performed three independent cold-shock experiments. In each, cells were grown at either 37°C or 27°C for 24 hours and collected and processed for immunoblotting analyses using anti-SUMO1 antibodies as described under Materials and Methods. 37°C: Samples grown at 37°C (normal conditions); 27°C: Samples grown at 27°C. In each case, lanes 1-3 represent samples from a different independent experiment.

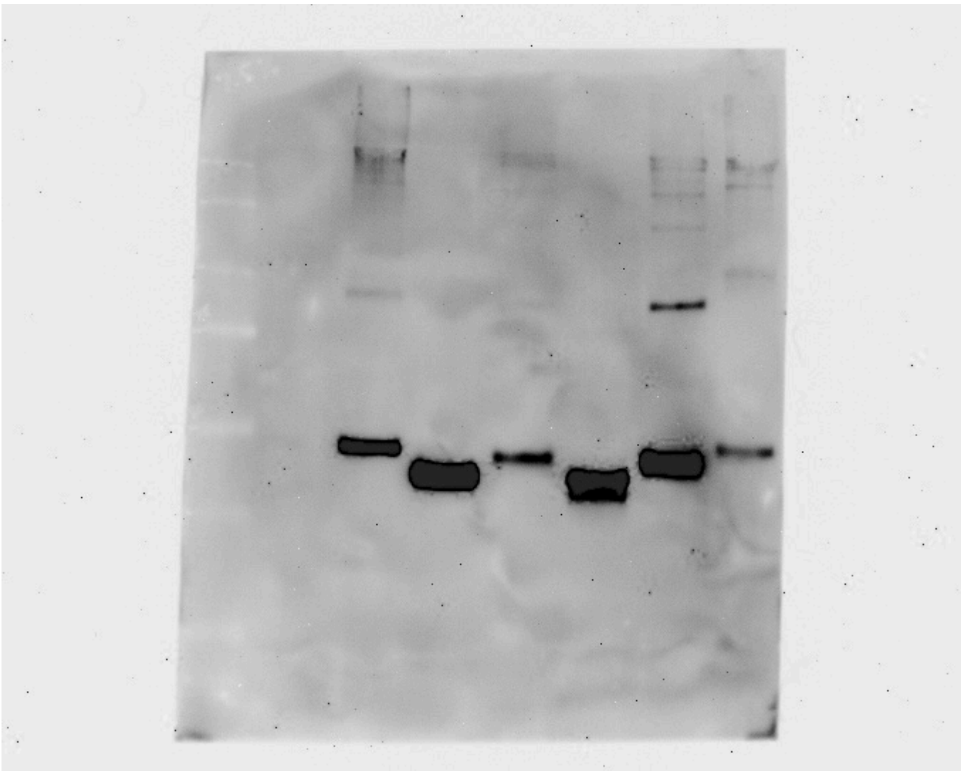

Supplementary Figure S6

HEK293A cells were transfected with expression constructs coding for the indicated His-S-YFP-tagged SUMO protein. At 24 h post-transfection, the transfected cells were collected in boiling 4x Sample Buffer and processed for SDS-PAGE and immunoblotting analysis. using antibodies against the S-tag, which is located between the His-tag (itself located near the N-terminus of the protein) and the YFP tag. The localization of free (unconjugated) and conjugated SUMO forms are indicated. 1, Cells transfected with an empty plasmid; 2, cells over-expressing His-S-YFP-SUMO1; 3, cells over-expressing His-S-YFP-SUMO1α; 4, cells over-expressing His-S-YFP-SUMO2; 5, cells over-expressing His-S-YFP-SUMO2α; 6, cells over-expressing His-S-YFP-SUMO3; 7, cells over-expressing His-S-YFP-SUMO3α. Notice the absence of truncated forms for all of the fusion proteins expressed, which indicates that the localization data provided in Figure 7 corresponds to the actual cellular distribution dictated by the corresponding SUMO protein. The expected molecular weights of the different fusion proteins are shown in the table provided. Notice also the lack of conjugated forms for His-S-YFP-SUMO1α and His-S-YFP-SUMO2α, which supports the conclusion that the alpha isoforms of SUMO1 and SUMO2 are not conjugatable (see Fig. 8D).
